# Application of Zr-MOFs based copper complex in synthesis of pyrazolo[3, 4-*b*]pyridine-5-carbonitriles via anomeric-based oxidation

**DOI:** 10.1038/s41598-023-34172-1

**Published:** 2023-06-09

**Authors:** Elham Tavakoli, Hassan Sepehrmansourie, Mahmoud Zarei, Mohammad Ali Zolfigol, Ardeshir Khazaei, Mohammad Ali As’Habi

**Affiliations:** 1 Department of Organic Chemistry, Faculty of Chemistry, Bu-Ali-Sina University, Hamedan, 6517838965 Iran; 2 Department of Chemistry, Faculty of Science, University of Qom, Qom, 37185-359 Iran; 3 Department of Phytochemistry, Medicinal Plant and Drugs Research Institute, Shahid Beheshti University, Evin, Tehran, 1983963113 Iran

**Keywords:** Catalyst synthesis, Heterogeneous catalysis, Organocatalysis

## Abstract

In this research article, Zr-MOFs based copper complex as a novel heterogeneous and porous catalyst was designed and prepared. The structure of catalyst has verified by various techniques such as FT-IR, XRD, SEM, *N*_2_ adsorption–desorption isotherms (BET), EDS, SEM-elemental mapping, TG and DTG analysis. UiO-66-NH_2_/TCT/2-amino-Py@Cu(OAc)_2_ was used as an efficient catalyst in the synthesis of pyrazolo[3,4-*b*]pyridine-5-carbonitrile derivatives. The aromatization of titled molecules is performed via a cooperative vinylogous anomeric-based oxidation both under air and inert atmospheres. The unique properties of the presented method are short reaction time, high yield, reusability of catalyst, synthesis of desired product under mild and green condition.

## Introduction

**Figure 1:**
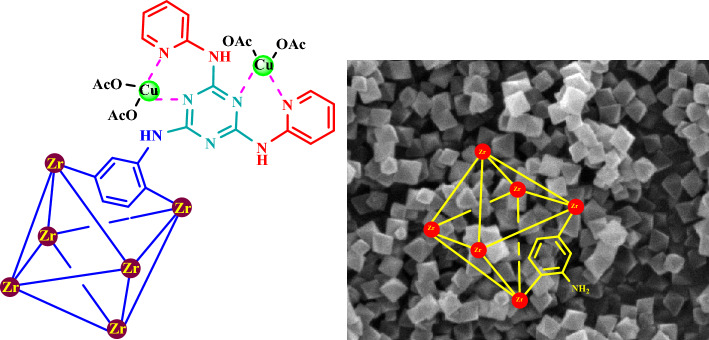
Structure and morphology of UiO-66(Zr)-NH_2_ as well as the final structure of a copper complex based on Zr-MOFs.

Nowadays, metal–organic frameworks as high surface areas materials are a new group of porous materials with potential applications such as gas storage and separation, drug delivery, sensors, batteries, supercapacitors as well as catalytic applications^1,2^. This framework is a class of organic–inorganic hybrid crystalline materials consisting of metallic nucleus that are linked by strong coordination bonds to organic ligands^3,4^. The different properties of these porous materials make them a good catalytic candidate for cross coupling, oxidation/reduction, and multicomponent reactions^5–10^. The post-modification method enhances catalytic performance and their variability. According this method, our research team reported a number of catalysts in the synthesis of organic compounds as biological active candidates^11–16^. Copper complex is widely used as catalysts in many organic reactions such as oxidation, cross coupling and catalytic organic reactions^17–19^. Recently, multicomponent reactions have investigated in the presence of palladium, nickel, copper, Fe, and Zr based catalytic systems^20–22^. In this report, a porous and heterogeneous catalyst based on Zr-MOFs with a copper complex is prepared. The simultaneous presence of copper and zirconium will enhance the catalytic application. This new system of porous complexes will lead to a new approach in the design and synthesis of catalysts. Figure 1 shows the final structure of the copper complex based on Zr-MOFs as well as the topology and structure of the UiO-66(Zr) grid.

**Figure 2:**
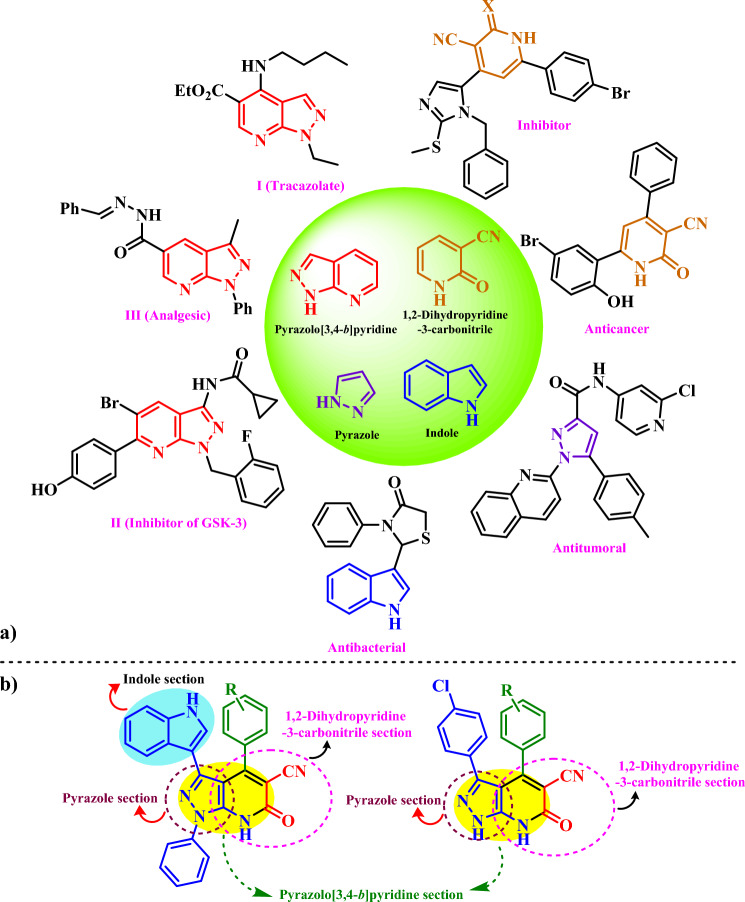
(**a**) The structure of compounds with medicinal and biological properties includes pyrazolo[3,4-*b*]pyridine, 1,2-dihydropyridine-3-carbonitrile, indole and pyrazole nucleus. (**b**) Target synthesized molecules with indole and pyrazole moieties.

Diversity of fused *N*-heterocycles such as pyrazolo[3,4-*b*]pyridine and 1,2-dihydropyridine-3-carbonitrile containing of indole and pyrazole moieties may be suitable candidates for biological and pharmacological studies^23–26^. These materials are suitable candidate for antimicrobial, anticancer, anticonvulsant, antifungal, HIV, anti-tumor, antioxidant, antihypertension and urinary incontinence treatment (Fig. 2a)^27–32^. The target synthesized molecules in this paper may be show biological properties due to the simultaneous presence of indole and pyrazole moieties (Fig. 2b).

**Figure 3:**
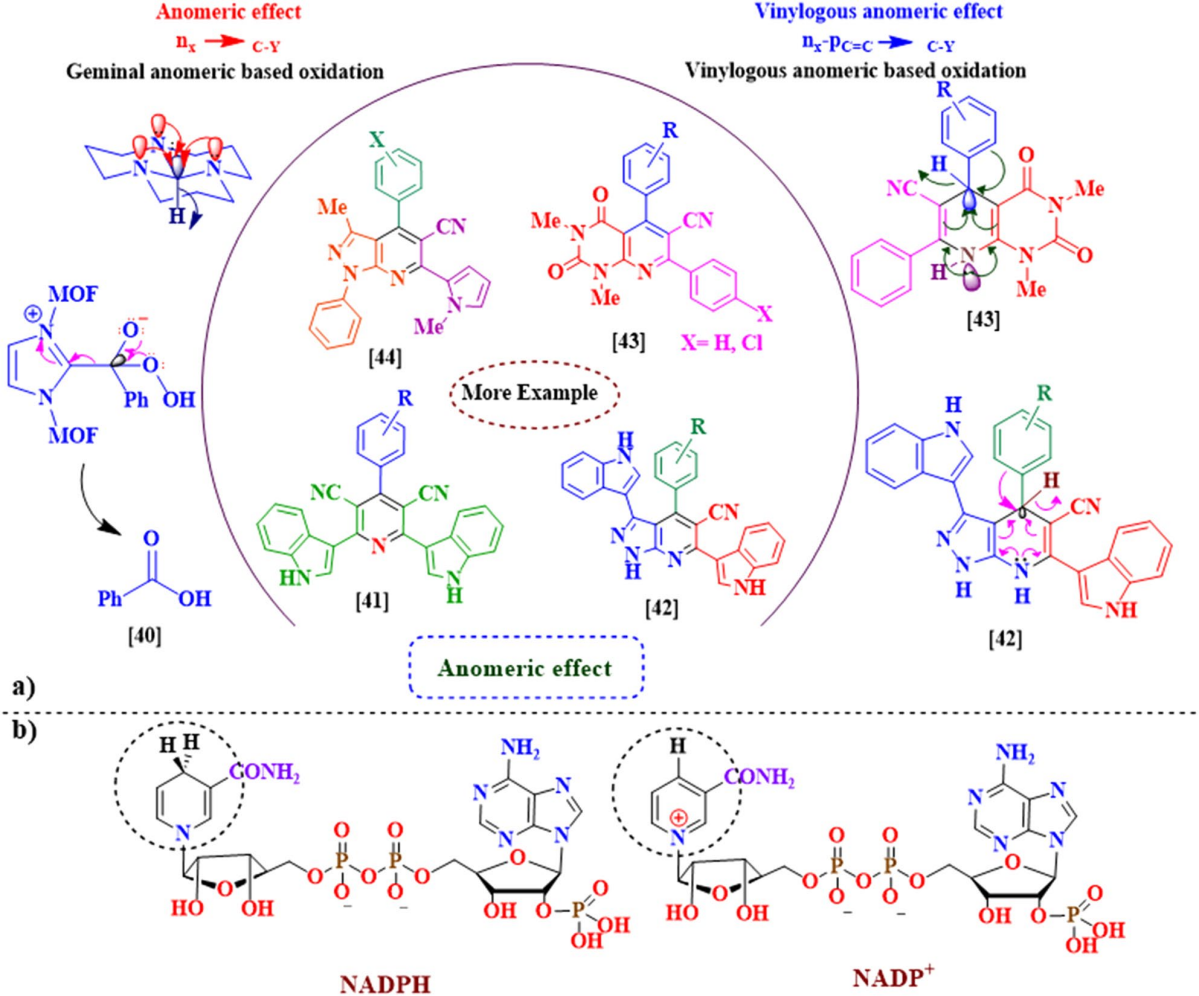
(**a**) The geminal versus vinylogous anomeric effect in organic synthesis. (**b**) The structures of NADPH/NADP^+^.

Anomeric effect (AE) as a fundamental example of stereoelectronic interactions has great educational and research applications^33–35^. It was discovered in 1955 by J. T. Edward in his studies on the carbohydrate chemistry^36^. The reported theory for the development of anomeric effect (AE) concept had been proposed that sharing the lone pair’s electrons of heteroatoms (X: N, O) to the anti-bonding orbital C–Y (n_X_ → σ^*^_C–Y_) weakened it (Fig. 3a). Stereoelectronic effects have also a major role in the oxidation–reduction of susceptible biological compounds such as NADPH/NADP^+^ (Fig. 3b)^37–39^. Recently, we and our coworkers have reviewed the role of the above-mentioned fundamental concepts comprehensively^34,35^.

**Figure 4:**
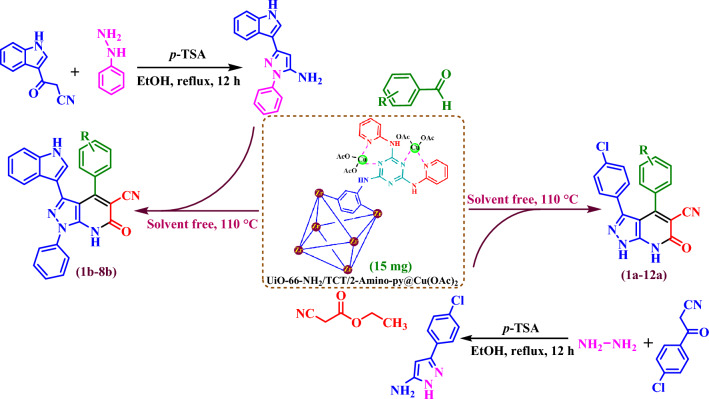
Preparation of pyrazolo[3,4-*b*]pyridine-5-carbonitriles using UiO-66-NH_2_/TCT/2-Amino-Py@Cu(OAc)_2_ as heterogeneous and porous catalyst.

According to the above-mentioned idea, we have architectonic and synthesis of a copper complex based on Zr-MOFs as a novel heterogeneous and porous catalyst. This porous catalyst was applied for the synthesis of pyrazolo[3,4-*b*]pyridine-5-carbonitriles by reaction of various aromatic aldehydes (bearing electron-donating and electron-withdrawing groups), ethyl cyanoacetate, 3-(1*H*-indol-3-yl)-1-phenyl-1*H*-pyrazol-5-amine or 3-(4-chlorophenyl)-1*H*-pyrazol-5-amine under solvent-free at 110 °C via a cooperative vinylogous anomeric-based oxidation (Fig. 4).

## Experimental section

### Materials

All materials and solvents used in this work such as zirconium chloride (ZrCl_4_, Merck, 99%), 2-amino terephthalic acid (NH_2_-BDC, Merck, 95%), 2-amino-pyridine (Merck, 95%), 2,4,6-trichloro-1,3,5-triazine (TCT, Merck, 98%), Cu(CH_3_COO)_2_) Merck, 95%), N(Et)_3_) Merck), EtOH (Merck, 99%), ethyl cyanoacetate (Merck, 98%), acetonitrile (Merck, 99% ), *p*-toluene sulfonic acid (Merck, 98.5%), aldehyde derivatives (Merck), hydrazine (Merck, 80% in H_2_O), phenyl hydrazine (Merck, 97%) and *N*,* N*-dimethylformamide (DMF, Aldrich, 99%) were obtained from commercial sources without further purification.

### Preparation of UiO-66-NH_2_/TCT/2-amino-Py@Cu(OAc)_2_ as heterogeneous and porous catalyst

**Figure 5:**
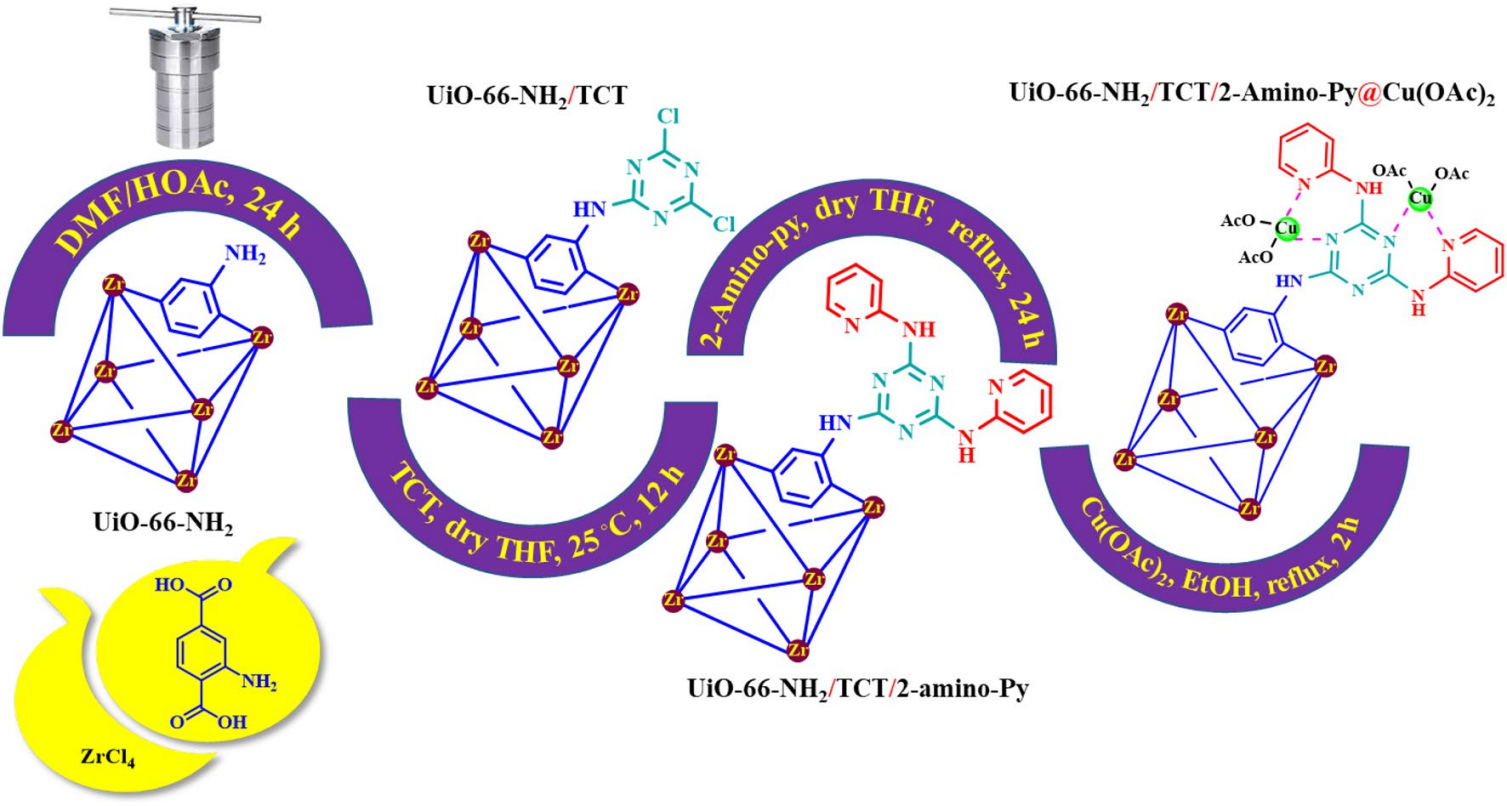
Synthesis Zr-MOFs based copper complex as a novel heterogeneous and porous catalyst.

Firstly, UiO-66-NH_2_ and UiO-66-NH_2_/TCT were synthesized according to the previously reports^45^. In a 50 mL round-bottom flask, UiO-66-NH_2_/TCT (0.5 g), 2-aminopyridine (7 mmol, 0.658 g), N(Et)_3_ (20 mol%, 0.02 g) and dry THF (25 mL) as a solvent were refluxed for 24 h. In the next step, solid mixture was separated by centrifuge (3000 rpm/min) and washed three times with ethanol and dried in a vacuum oven at 60 °C for 12 h^46,47^. In the following, in a 25 mL round-bottom flask, a mixture of UiO-66-NH_2_/TCT/2-amino-Py (0.5 g) and Cu(CH_3_COO)_2_ (0.2 mmol, 0.036 g) were stirred in ethanol (20 mL) as a solvent in room temperature for 2 h. Then, UiO-66-NH_2_/TCT/2-amino-Py@Cu(OAc)_2_ was filtered using a centrifuge (3000 rpm/min) and dried under vacuum at 60 °C to grow a copper complex based on Zr-MOFs as a novel heterogeneous and porous catalyst (Fig. 5).

### General method for the preparation of new pyrazolo[3,4-*b*]pyridine-5-carbonitriles

The first, raw materials such as 3-(1*H*-indol-3-yl)-1-phenyl-1*H*-pyrazol-5-amine **(1)** and 3-(4-chlorophenyl)-1*H*-pyrazol-5-amine **(2)** were prepared according to the previously reported (Fig. 4)^48–52^. In the following, in a 10 mL round-bottomed flask, a mixture of aromatic aldehydes (1 mmol), ethyl cyanoacetate (1 mmol, 0.113 g) and **(1)** or/and**(2)** in percent of UiO-66-NH2/TCT/2-amino-Py@Cu(OAc)_2_ (15 mg) as catalyst were stirred under solvent-free condition at 110 °C. The progress and completion of the reaction was monitored by using TLC technique. Then the reaction mixture was allowed to cooled up to room temperature. The reaction mixture was dissolved in hot ethanol (20 mL) to separate the catalyst by using centrifugation (4000 rpm/min). The desired products (1a–12a) were washed with acetone/ethanol and collected by simple filtration and (1b–8b). Finally, the crude products were purified by column chromatography (Fig. 4).

## Results and discussion

Since the role of the anomeric effect can be found in the course of synthesis of various organic compounds^53,54^, herein, we decided to synthesize new compounds via an anomeric supporting mechanism. On the other hand, the importance of developing new catalysts for chemical reactions increased our motivation to produce new porous catalysts. Creating a copper complex based on metal–organic frameworks creates a new approach to the preparation of heterogeneous catalysts. The structure of UiO-66-NH_2_/TCT/2-amino-Py@Cu(OAc)_2_ as a porous and heterogeneous catalyst was completely identified using various techniques such as FT-IR, XRD, SEM, *N*_2_ adsorption–desorption isotherms (BET), BJH, EDS, SEM-elemental mapping, TG and DTG. The UiO-66-NH_2_/TCT/2-amino-Py@Cu(OAc)_2_ was used for prepareing new pyrazolo[3,4-*b*]pyridine-5-carbonitriles. These compounds may be had biological and medicinal applications due to the presence of indole and pyrazole moieties. The structure of the synthesized compounds was confirmed using FT-IR, ^1^H-NMR, ^13^C-NMR and melting point techniques. This report describes all the experiments package, including the synthesis of catalyst, the optimization and mechanism of the reaction via anomeric-based oxidation pathway for the aromatization of the mentioned molecules under air and neutral atmosphere.

**Figure 6:**
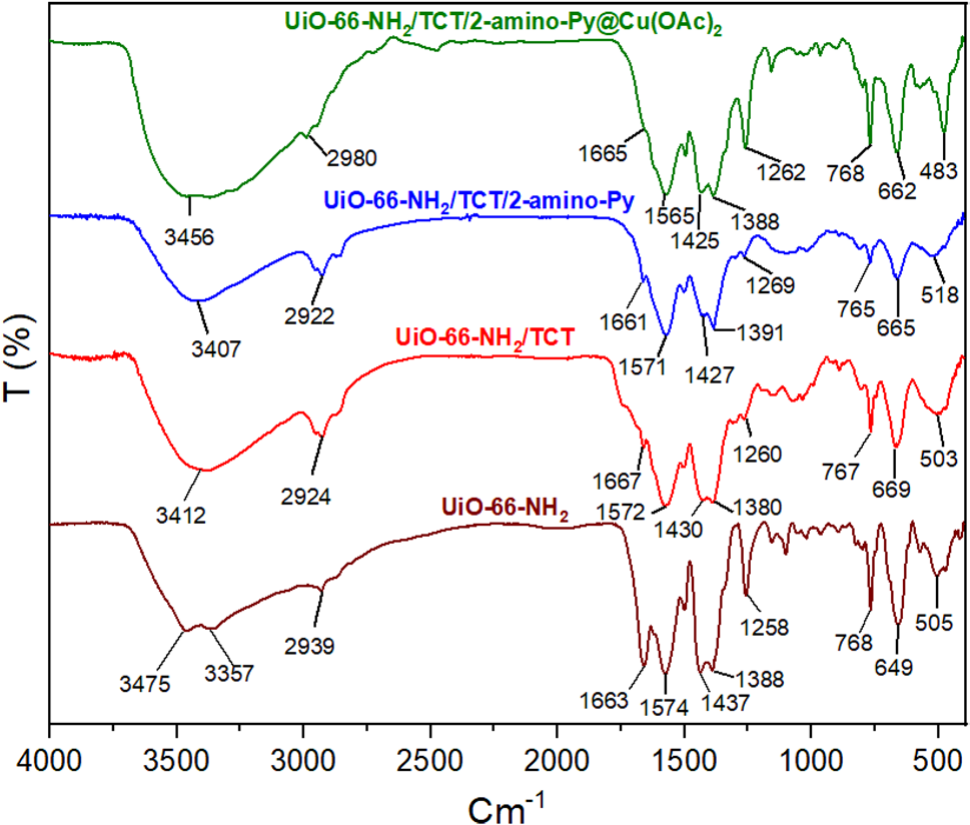
FT-IR spectra of catalyst and starting materials.

The FT-IR spectra of UiO-66-NH_2_/TCT/2-amino-Py@Cu(OAc)_2_ as a catalyst and starting materials were shown in Fig. 6. The two peaks at 3475 and 3357 cm^−1^ of NH_2_ functional groups are represented synthesis of UiO-66-NH_2_^45^. Also, the absorption peaks at 2800–3000 cm^−1^ are related to aromatic C–H and C=C stretches bands. The addition of different compounds during the catalyst synthesis steps results in changes in the spectra that indicate a change in structure.

**Figure 7:**
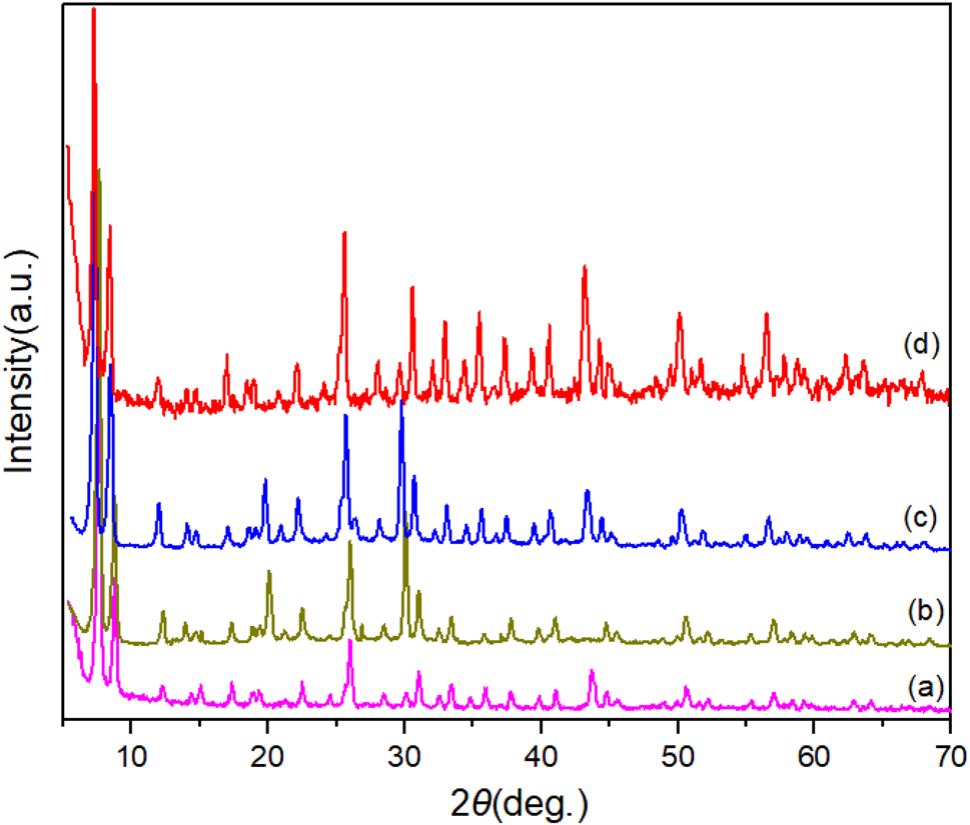
Comparison XRD pattern of (**a**) UiO-66-NH_2,_ (**b**) UiO-66-NH_2_/TCT (**c**) UiO-66-NH_2_/TCT/2-amino-Py and (**d**) UiO-66-NH_2_/TCT/2-Amino-Py@Cu(OAc)_2_ a copper complex based on Zr-MOFs as a novel heterogeneous and porous catalyst.

The XRD pattern of different stages of materials and catalyst synthesis was compared (Fig. 7). The XRD pattern of UiO-66-NH_2_ was identical to the previously reported data^45^. The last stage of copper complex based on Zr-MOFs has been proved by appearing of peaks. Also, below the peak at 2*θ* < 10, indicating the structure of the crystal plates of the various phases has suitable stability.

**Figure 8:**
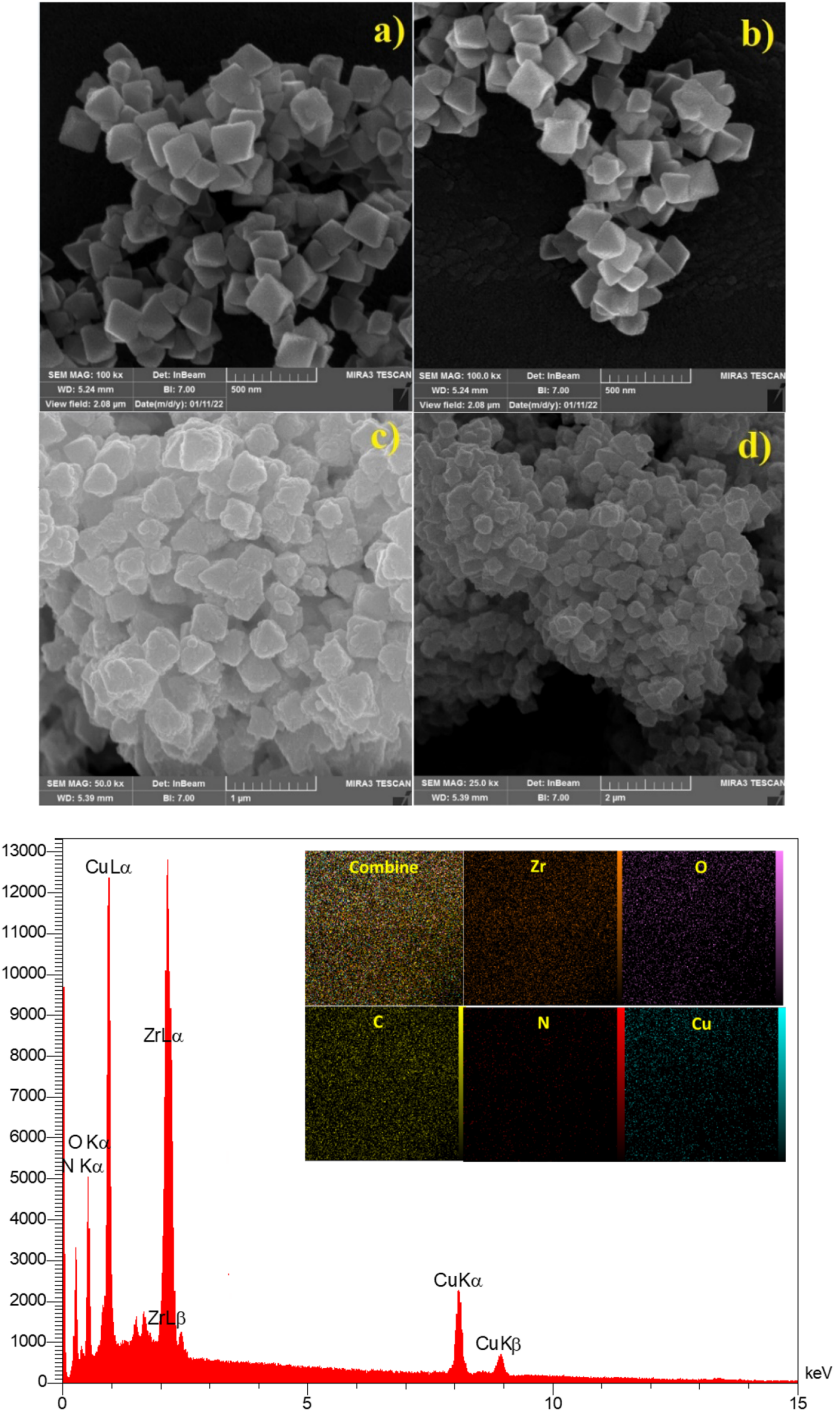
(**a**) Scanning electron microscope (SEM) images of UiO-66-NH_2_ (**a**,**b**) and UiO-66-NH_2_/TCT/2-amino-Py@Cu(OAc)_2_ (**c**,**d**). (**b**) EDX spectroscopy and elemental mapping analysis of UiO-66-NH_2_/TCT/2-amino-Py@ Cu(OAc)_2_ a copper complex based on Zr-MOFs as a novel heterogeneous and porous catalyst.

The morphology of UiO-66-NH_2_ and UiO-66-NH_2_/TCT/2-amino-Py@Cu(OAc)_2_ was also studied by scanning electron microscopy (SEM) technique (Fig. 8a). As shown in Fig. 8a, morphology of catalyst particles is tetrahedral which is in good condition and not completely stacked. Also, the morphology of UiO-66-NH_2_ is stable after post-modification. Elemental mapping analysis shows Zr, N, O, C and Cu atoms which were confirmed in the structure of UiO-66-NH_2_/TCT/2-amino-Py@Cu(OAc)_2_ (Fig. 8b). Furthermore, the well-dispersed distribution of elements in the UiO-66-NH_2_/TCT/2-amino-Py@Cu(OAc)_2_ was determined and verified by elemental mapping analysis (Fig. 8b).

**Figure 9:**
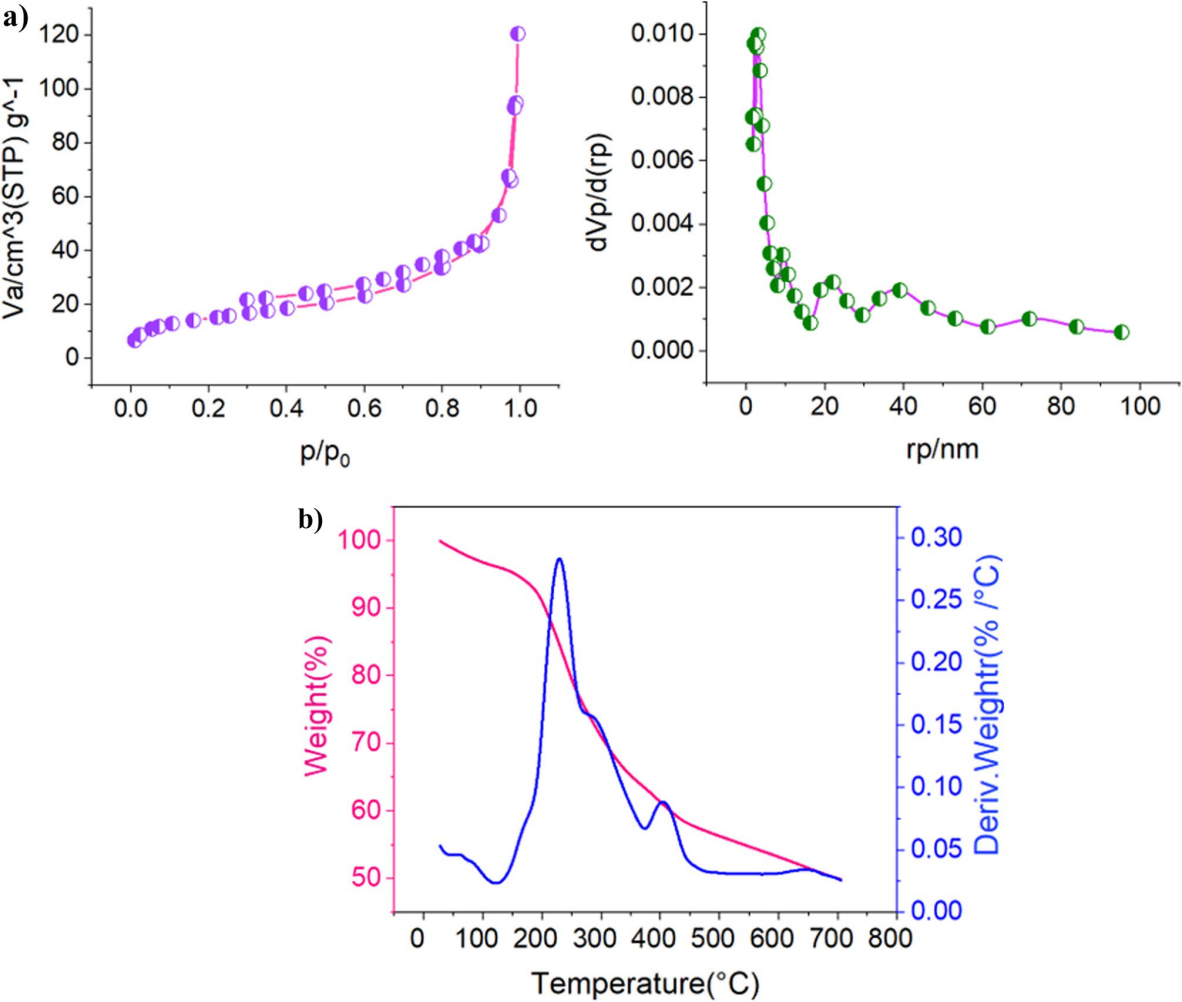
(**a**) *N*_2_ adsorption–desorption isotherms and the pore size distribution of UiO-66-NH_2_/TCT/2-amino-Py@Cu(OAc)_2_. (**b**) Thermal gravimetric (TG) and derivative thermal gravimetric (DTG) analysis of UiO-66-NH_2_/TCT/2-amino-Py@Cu(OAc)_2_ as a novel heterogeneous and porous catalyst.

In another searching, the textural properties of UiO-66-NH_2_/TCT/2-amino-Py@Cu(OAc)_2_ were studied by *N*_2_ adsorption–desorption isotherms (Fig. 9a). Based on the obtained results, the area calculated based on the BET equation, the total pore volume 115 m^2^ g^−1^ and 0.1523 cm^3^ g^−1^ respectively. The pore size distribution of UiO-66-NH_2_/TCT/2-amino-Py@Cu(OAc)_2_ based on BJH method is shown in (Fig. 9a). The mean pore diameter for the catalyst is 8.48 nm. The presence of a suitable surface area, as well as the size of catalyst cavities can be a major reason for the high efficiency at the synthesis of pyrazolo[3,4-*b*]pyridine-5-carbonitriles. The thermal gravimetric (TG) and derivative thermal gravimetric (DTG) analysis of UiO-66-NH_2_/TCT/2-amino-Py@Cu(OAc)_2_ was shown in Fig. 9b. According to this diagram, several failures due to the separation of the copper complex and organic compounds of Zr-MOFs are shown. The diagram shows that the synthesized catalyst is stable up to 240 °C.

After confirming the structure of a copper complex based on Zr-MOFs, we attempted to evaluate its catalytic performance for the synthesis of various new pyrazolo[3,4-*b*]pyridine-5-carbonitriles. For this purpose, we selected the reaction between 4-chloro-benzaldehyde (1 mmol, 0.140 g), ethyl cyanoacetate (1 mmol, 0.113 g) and 3-(4-chlorophenyl)-1*H*-pyrazol-5-amine (1 mmol, 0.193 g) as a model reaction. In order to select suitable conditions, the model of reaction was evaluated using various solvents, different temperatures and amounts of catalysts. The results are shown in Table 1. According to the presented data in Table 1, the best of choice for the synthesis of pyrazolo[3,4-*b*]pyridine-5-carbonitriles was achieved in percent of UiO-66-NH_2_/TCT/2-amino-Py@Cu(OAc)_2_ (15 mg) as catalyst under solvent-free conditions at 110 °C.

**Table 1:** Effect of different amounts of catalyst, temperature and solvent on the synthesis of pyrazolo[3,4-*b*]pyridine-5-carbonitriles.


Entry	Catalyst (mg)	Temp. (°C)	Solvent	Time (min.)	Yield (%)
1	20	110	–	120	85
**2**	**15**	**110**	**–**	**120**	**85**
3	10	110	–	120	76
4	5	110	–	120	70
5	–	110	–	120	43
6	15	Reflux	EtOAc	300	45
7	15	Reflux	EtOH	240	60
8	15	Reflux	MeOH	240	45
9	15	Reflux	CH_3_CN	300	55
10	15	Reflux	H_2_O	300	20
11	15	Reflux	H_2_O/EtOH	300	60
12	15	Reflux	H_2_O/CH_3_CN	360	Trace
13	15	Reflux	CHCl_3_	360	30
14	15	Reflux	Acetone	300	Trace
15	15	70	–	120	60
16	15	90	–	120	73
17	15	110	–	30	40
18	15	110	–	60	73

Significant values are in [bold].

**Figure 10:**
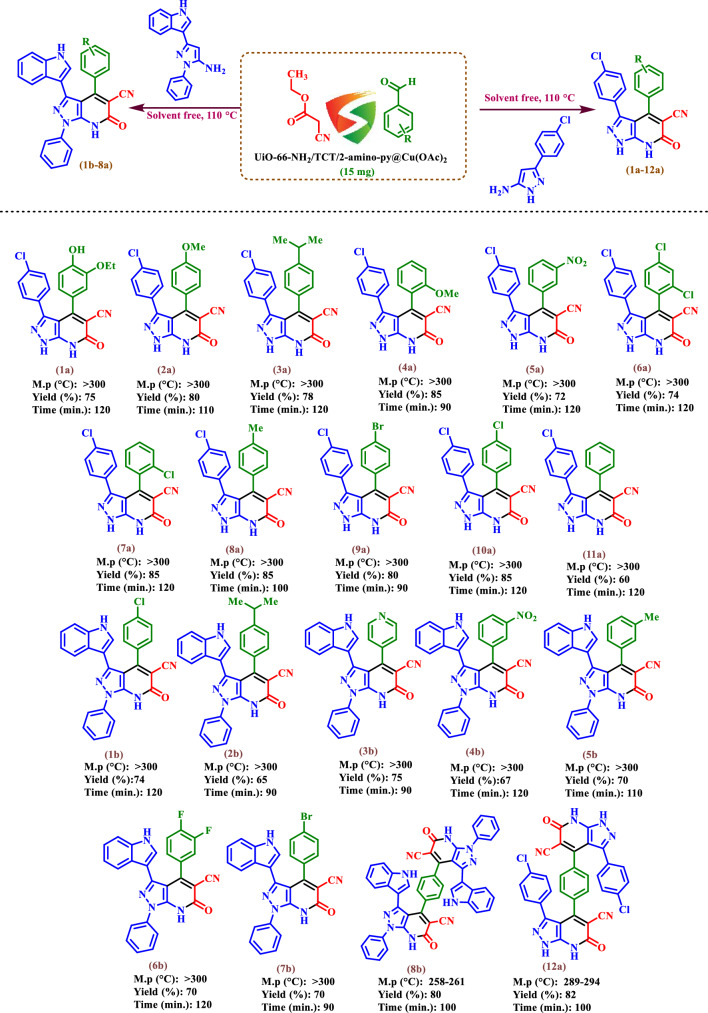
Synthesis of pyrazolo[3,4-*b*]pyridine-5-carbonitriles using UiO-66-NH_2_/TCT/2-amino-Py@Cu a copper complex based on Zr-MOFs as a novel heterogeneous and porous catalyst.

After selecting the optimal conditions for the synthesis of 3,4-bis(4-chlorophenyl)-6-oxo-6,7-dihydro-1*H*-pyrazolo[3,4-*b*]pyridine-5-carbonitrile (**1a**), a wide range of aromatic aldehydes including electron withdrawing, electron releasing and heterocyclic rings were tested for obtaining of desired products (Fig. 10). As shown in Fig. 10, the obtained results indicated that UiO-66-NH_2_/TCT/2-amino-Py@Cu(OAc)_2_ is appropriate for the preparation of target molecules in high to excellent yields (60–85%) with relatively short reaction times (90–120 min.).

To evaluate the performance of UiO-66-NH_2_/TCT/2-amino-Py@Cu(OAc)_2_ as a catalyst in comparison to those other catalysts for the preparation pyrazolo[3,4-*b*]pyridine-5-carbonitriles, we have used various homogeneous and heterogeneous catalysts and previous stages of the final catalyst for the condensation reaction 4-chloro-benzaldehyde (1 mmol, 0.140 g), ethyl cyanoacetate (1 mmol, 0.113 g) and 3-(4-chlorophenyl)-1*H*-pyrazol-5-amine (1 mmol, 0.193 g) as a model reaction in Table 2. As shown the obtained data in the Table 2, UiO-66-NH_2_/TCT/2-amino-Py@Cu(OAc)_2_ is the best catalyst for the synthesis of pyrazolo[3,4-*b*]pyridine-5-carbonitrile derivatives.

**Table 2:** Evaluation of various catalyst for the synthesis of pyrazolo[3,4-*b*]pyridine-5-carbonitriles with UiO-66-NH_2_/TCT/2-Amino-py@cu.

Entry	Catalyst	Amount of catalyst	Time (min.)	Yield (%)
**1**	**UiO-66-NH**_**2**_**/TCT/2-amino-py@Cu(OAc)**_**2**_** (This work)**	**15 (mg)**	**120**	**85**
2	UiO-66-NH_2_^45^	15 (mg)	180	22
3	UiO-66-NH_2_/TCT	15 (mg)	180	Trace
4	UiO-66-NH_2_/TCT/2-amino-py	15 (mg)	150	38
5	Cu(OAc)_2_	15 (mol%)	180	35
6	ZrCl_4_	15 (mol%)	180	–
7	GTBSA^55^	15 (mg)	120	74
8	N(Et)_3_	15 (mol%)	180	76
9	[PVI-SO_3_H]Cl^56^	15 (mol%)	150	65
10	*p*-TSA	15 (mol%)	120	45
11	NaOH	15 (mol%)	100	66
12	Pipyridine	15 (mol%)	120	80
13	MIL(100)(Cr)/NHEtN(CH_2_PO_3_H_2_)_2_^11,12^	15 (mg)	100	74
14	KOH	15 (mol%)	120	70
15	[Py-SO_3_H]Cl^57^	15 (mol%)	120	63
16	SSA^58,59^	15 (mol%)	180	56

Significant values are in [bold].

**Figure 11:**
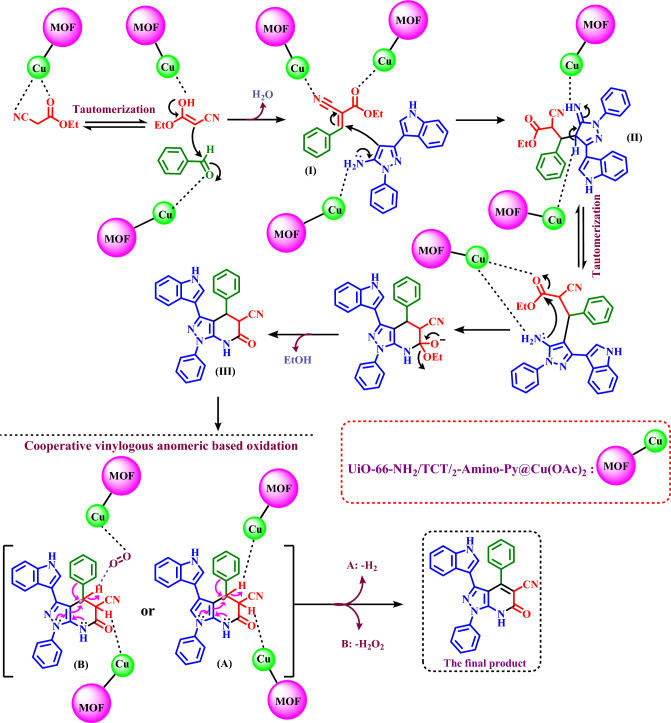
The proposed mechanism for the synthesis of pyrazolo[3,4-*b*]pyridine-5-carbonitriles using UiO-66-NH_2_/TCT/2-Amino-Py@Cu(OAc)_2_.

Suggested mechanism for the synthesis of pyrazolo[3,4-*b*]pyridine-5-carbonitriles using UiO-66-NH_2_/TCT/2-amino-Py@Cu(OAc)_2_ as a heterogeneous and porous catalyst was shown in Fig. 11. At the first step, ethyl cyanoacetate  is converted to enolate form and react with activated aldehyde to produce intermediate (**I**) by losing one molecule of H_2_O. In the following, (3-(1*H*-indol-3-yl)-1-phenyl-1*H*-pyrazol-5-amine **(1)** and/or 3-(4-chlorophenyl)-1*H*-pyrazol-5-amine **(2)** attack to intermediate (**I**) as a Michael acceptor created intermediate (**II**). In the next step, intermediate (**II**) is converted to intermediate (**III**) through tautomerization and intramolecular cyclization. Finally, the intermediate (**III**) converts to their corresponding derivatives via a cooperative vinylogous anomeric based oxidation and releases one molecule of hydrogen (–H_2_) and/or hydrogen peroxide (–H_2_O_2_) molecules^26,60,61^. The obtained results of the reaction model under argon, nitrogen and oxygen atmospheres are similar which are verified the presented mechanism. The term cooperative is used when more than one lone pair of electrons and other donors are sharing the anti-bonding orbitals of one acceptor bond (n_N_ → σ^*^_C–H_). The simultaneous cooperative sharing of electrons from donors into the anti-bonding orbitals of the C–H bond is a major driving force for hydride releasing (n_N_ → σ^*^_C–X_).

**Figure 12:**
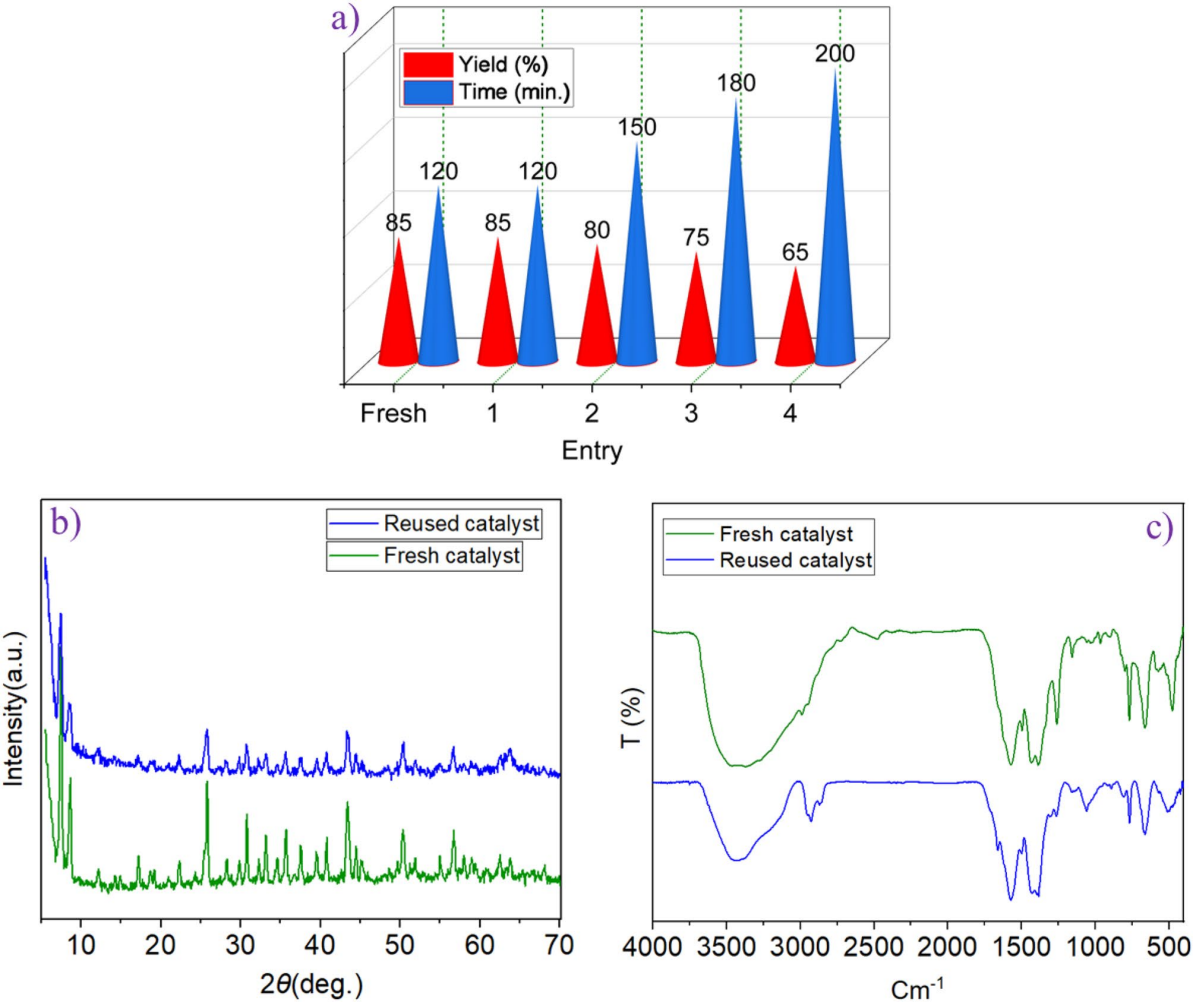
(**a**) Recyclability of catalyst for the synthesis of pyrazolo[3,4-*b*]pyridine-5-carbonitriles. Comparison (**b**) XRD, (**c**) FT-IR of reused and fresh catalyst.

To prove the recyclability of the presented catalyst, we tested model reaction under the optimal reaction conditions in the previously section. The results of Fig. 12a show that the UiO-66-NH_2_/TCT/2-amino-Py@Cu(OAc)_2_ as a catalyst can be reused up to 4 times without noticeable changes in its catalytic activity. This performance indicates the high stability of the copper complex created on the Zr-MOFs as a heterogeneous and porous catalyst. To prove the stability of the catalyst structure, the recovered catalyst was evaluated by FT-IR and XRD analysis. The results are shown in Fig. 12b and c. According to the results, there have been not many changes in the catalyst structure, indicating the stability of the catalyst. Also, to investigate the heterogeneous nature of the protocols and Cu leaching, ICP results proved that no Zr and Cu leaching was detected in the filtrate (Zr: 2.41 × 10^−6^ and Cu: 2.03 × 10^−5^ mol/g respectively) upon reaction completion, which indicates the high stability of the prepared catalyst.

## Conclusions

In summary, Zr-MOFs based copper complex was introduced. At this catalyst, copper was supported on the surface of metal–organic frameworks as a new porous complex. Proper stability and morphology of the presented catalyst can create a new approach in the preparation of porous and heterogeneous catalysts. Catalytic performance of UiO-66-NH_2_/TCT/2-amino-Py@Cu(OAc)_2_ was demonstrated in the synthesis of new pyrazolo[3,4-*b*]pyridine-5-carbonitriles via anomeric based oxidation concept. These compounds can have biological and medicinal applications due to the presence of indole and pyrazole nucleus. High efficiency of products and gentle green conditions are other features of the products synthesized using this new porous and heterogeneous catalyst.

## Supplementary Information

Supplementary Information.

## Data Availability

The datasets used and/or analyzed during the current study available from the corresponding author on reasonable request.
