# Supplementary material for: Application of Zr-MOFs based copper complex in synthesis of pyrazolo[3, 4-b]pyridine-5-carbonitriles via anomeric-based oxidation
Source: Sci Rep. 2023 Jun 9;13:9388. doi: 10.1038/s41598-023-34172-1 (PMC10256735; doi:10.1038/s41598-023-34172-1)
Supplement: Supplementary file 1 — Supplementary Information. [file 41598_2023_34172_MOESM1_ESM.docx]

**Application of Zr-MOFs based copper complex in synthesis of pyrazolo[3, 4-*b*]pyridine-5-carbonitriles via anomeric-based oxidation**

Elham Tavakoli,^a^ Hassan Sepehrmansourie,^a^ Mahmoud Zarei,^*b^ Mohammad Ali Zolfigol^*a^, Ardeshir Khazaei^*a^ and Mohammad Ali As’Habi ^c^

^a^ Department of Organic Chemistry, Faculty of Chemistry, Bu-Ali-Sina University, 6517838965, Hamedan, Iran., E-mail: [zolfi@basu.ac.ir](mailto:zolfi@basu.ac.ir), [mzolfigol@yahoo.com](mailto:m.zolfigol@yahoo.com) (M. A. Zolfigol) or Khazaei_1326@yahoo.com (A. Khazaei).

^b^ Department of Chemistry, Faculty of Science, University of Qom, Qom, 37185-359, Iran. E- Mail: mahmoud8103@yahoo.com (M. Zarei).

^c^ Department of Phytochemistry, Medicinal Plant and Drugs research Institute, Shahid Beheshti University, Evin, Tehran, 1983963113, Iran.

# Spectral data of compounds

**3-(4-Chlorophenyl)-4-(3-ethoxy-4-hydroxyphenyl)-6-oxo-6,7-dihydro-1*H*-pyrazolo[3,4-*b*]pyridine-5-carbonitrile (1a).**

White solid; M.p: >300 ˚C; (*n-*Hexane: Ethyl acetate 2:8). FT-IR (KBr, cm^-1^): 3433, 2978, 2846, 2229, 1638, 1595. ^1^H NMR (400 MHz, DMSO*-d_6_*) δ_ppm_ 9.80 (s, 3H), 7.08 (d, *J* = 8.6 Hz, 2H), 6.93 (d, *J* = 8.6 Hz, 2H), 6.71 (s, 2H), 6.51 (s, 1H), 2.99 (t, *J* = 3.9 Hz, 2H), 1.14 (t, *J* = 7.0 Hz, 3H). ^13^C NMR (101 MHz, DMSO*-d_6_*) δ_ppm_ 161.6, 147.6, 146.0, 135.8, 131.8, 130.1, 128.9, 126.3, 121.7, 114.9, 114.8, 105.4, 63.6, 23.2. Mass spectrum calcd for C_21_H_15_ClN_4_O_3_: 406.0833, found: 407.0837.

**D_2_O exchange 3-(4-chlorophenyl)-4-(3-ethoxy-4-hydroxyphenyl)-6-oxo-6,7-dihydro-1*H*-pyrazolo[3,4-*b*]pyridine-5-carbonitrile (1a).**

^1^H NMR (400 MHz, DMSO*-d_6_*) δ_ppm_ 9.80 (s, 3H), 7.08 (d, *J* = 8.6 Hz, 2H), 6.93 (d, *J* = 8.6 Hz, 2H), 6.71 (s, 2H), 6.51 (s, 1H), 2.99 (t, *J* = 3.9 Hz, 2H), 1.14 (t, *J* = 7.0 Hz, 3H). ^1^H NMR (400 MHz, D_2_O) δ_ppm_ 6.81 (dd, *J* = 7.9, 3.4 Hz, 2H), 6.66 (dd, *J* = 8.0, 3.2 Hz, 2H), 6.45 (s, 2H), 6.22 (s, 1H), 2.72 (t, *J* = 4.4 Hz, 2H), 0.88 (t, *J* = 7.0 Hz, 3H).

**3-(4-Chlorophenyl)-4-(4-methoxyphenyl)-6-oxo-6,7-dihydro-1*H*-pyrazolo[3,4-*b*]pyridine-5-carbonitrile (2a).**

White solid; M.p: >300 ˚C; (*n-*Hexane: Ethyl acetate 2:8). FT-IR (KBr, cm^-1^): 3459, 2214, 1647, 1511, ^1^H NMR (400 MHz, DMSO*-d_6_*) δ_ppm_ 9.45 (s, 2H), 8.03 (d, *J* = 8.6 Hz, 2H), 7.42 (d, *J* = 8.3 Hz, 2H), 6.95 (dd, *J* = 72.3, 8.3 Hz, 4H), 3.08 (s, 3H). ^13^C NMR (101 MHz, DMSO*-d_6_*) δ_ppm_ 167.0, 154.7, 146.9, 141.0, 134.5, 132.2, 131.6, 130.6, 130.2, 128.6, 128.0, 127.7, 126.9, 121.5, 116.1, 106.0, 67.4.

**3-(4-Chlorophenyl)-4-(4-isopropylphenyl)-6-oxo-6,7-dihydro-1*H*-pyrazolo[3,4-*b*]pyridine-5-carbonitrile (3a)****.**

White solid; M.p: >300 ˚C; (*n-*Hexane: Ethyl acetate 2:8). FT-IR (KBr, cm^-1^): 3182, 3109, 2961, 2866, 2218, 1584, 1514. ^1^H NMR (400 MHz, DMSO*-d_6_*) δ_ppm_ 10.11 (s, 2H), 7-7.4 (m, 4H), 6.97 (d, *J* = 8.6 Hz, 2H), 6.81 (d, *J* = 8.5 Hz, 2H), 2.84 (h, *J* = 6.8 Hz, 1H), 1.19 (d, *J* = 6.9 Hz, 6H). ^13^C NMR (101 MHz, DMSO*-d_6_*) δ_ppm_ 168.8, 155.0, 151.5, 148.9, 144.1, 133.0, 132.2, 131.4, 129.9, 128.5, 127.0, 125.6, 119.7, 102.2, 95.0, 33.3, 23.8, 22.8. Mass spectrum calcd for C_22_H_17_ClN_4_O: 388.1091, found: 389.0661.

**3-(4-Chlorophenyl)-4-(2-methoxyphenyl)-6-oxo-6,7-dihydro-1*H*-pyrazolo[3,4-*b*]pyridine-5-carbonitrile (4a).**

White solid; M.p: >300 ˚C; (*n-*Hexane: Ethyl acetate 2:8). FT-IR (KBr, cm^-1^): 3428, 3020, 2839, 2209, 1651, 1602. ^1^H NMR (400 MHz, DMSO*-d_6_*) δ_ppm_ 12.90 (s, 2H), 7.35 (t, *J* = 7.9 Hz, 1H), 7.24 (d, *J* = 7.5 Hz, 1H), 7.12 (d, *J* = 8.3 Hz, 2H), 6.98 – 6.87 (m, 3H), 6.75 (d, *J* = 8.5 Hz, 1H), 3.33 (s, 3H). ^13^C NMR (101 MHz, DMSO*-d_6_*) δ_ppm_ 161.9, 155.5, 153.3, 149.4, 142.3, 133.2, 131.3, 129.9, 129.6, 128.0, 127.4, 122.8, 120.3, 116.6, 111.0, 103.0, 55.4. Mass spectrum calcd for C_20_H_13_ClN_4_O_2_: 376.0727, found: 376.9444.

**3-(4-Chlorophenyl)-4-(3-nitrophenyl)-6-oxo-6,7-dihydro-1*H*-pyrazolo[3,4-*b*]pyridine-5-carbonitrile (5a).**

White solid; M.p: >300 ˚C; (*n-*Hexane: Ethyl acetate 2:8). FT-IR (KBr, cm^-1^): 3442, 2983, 2678, 2480, 2211, 1594, 1527. ^1^H NMR (400 MHz, DMSO*-d_6_*) δ_ppm_ 12.35 (s, 2H), 8.19 (d, *J* = 8.2 Hz, 1H), 7.86 (s, 1H), 7.77 (d, *J* = 7.9 Hz, 1H), 7.60 (t, *J* = 8.0 Hz, 1H), 7.06 (d, *J* = 8.5 Hz, 2H), 6.90 (d, *J* = 8.6 Hz, 2H). ^13^C NMR (101 MHz, DMSO*-d_6_*) δ_ppm_ 163.4, 151.9, 151.1, 146.9, 142.4, 135.3, 135.2, 133.1, 130.5, 129.9, 129.0, 127.5, 123.8, 123.7, 117.1, 102.7. Mass spectrum calcd for C_19_H_10_ClN_5_O_3_: 391.0427, found: 392.0038.

**3-(4-Chlorophenyl)-4-(2,4-dichlorophenyl)-6-oxo-6,7-dihydro-1*H*-pyrazolo[3,4-*b*]pyridine-5-carbonitrile (6a).**

White solid; M.p: >300 ˚C; (*n-*Hexane: Ethyl acetate 2:8). FT-IR (KBr, cm^-1^): 3198, 2237, 1651, 1591. ^1^H NMR (400 MHz, DMSO*-d_6_*) δ_ppm_ 12.74 (s, 2H), 7.53 (s, 1H), 7.40 (d, 2H), 7.17 (d, *J* = 8.5 Hz, 2H), 6.96 (d, *J* = 8.6 Hz, 2H). ^13^C NMR (101 MHz, DMSO*-d_6_*) δ_ppm_ 150.4, 135.1, 133.4, 132.1, 131.7, 130.2, 128.7, 128.1, 127.6, 127.4, 116.3. Mass spectrum calcd for C_19_H_9_Cl_3_N_4_O: 413.9842, found: 414.9333.

**4-(2-Chlorophenyl)-3-(4-chlorophenyl)-6-oxo-6,7-dihydro-1*H*-pyrazolo[3,4-*b*]pyridine-5-carbonitrile (7a).**

White solid; M.p: >300 ˚C; (*n-*Hexane: Ethyl acetate 2:8). FT-IR (KBr, cm^-1^): 3030, 2811, 2213, 1657, 1595, 1566. ^1^H NMR (400 MHz, DMSO*-d_6_*) δ_ppm_ 12.72 (s, 2H), 7.39 – 7.28 (m, 4H), 7.10 (d, *J* = 8.6 Hz, 2H), 6.94 (d, *J* = 8.5 Hz, 2H). ^13^C NMR (101 MHz, DMSO*-d_6_*) δ_ppm_ 164.9, 157.9, 133.9, 132.7, 131.1, 130.7, 130.4, 129.8, 129.2, 127.4, 127.1, 117.4, 109.2, 102.4. Mass spectrum calcd for C_19_H_10_Cl_2_N_4_O: 380.0232, found: 381.0398.

**3-(4-Chlorophenyl)-6-oxo-4-(*p*-tolyl)-6,7-dihydro-1*H*-pyrazolo[3,4-*b*]pyridine-5-carbonitrile (8a).**

White solid; M.p: >300 ˚C; (*n-*Hexane: Ethyl acetate 2:8). FT-IR (KBr, cm^-1^): 3030, 2835, 2213, 1662, 1590. ^1^H NMR (400 MHz, DMSO*-d_6_*) δ_ppm_ 12.88 (s, 2H), 7.09 (d, *J* = 8.6 Hz, 2H), 7.06 – 6.99 (m, 4H), 6.89 (d, *J* = 8.6 Hz, 2H), 2.28 (s, 3H). ^13^C NMR (101 MHz, DMSO*-d_6_*) δ_ppm_ 162.3, 151.7, 142.8, 139.2, 133.9, 132.5, 131.5, 130.2, 128.3, 127.3, 121.3, 118.0, 102.6, 20.7. Mass spectrum calcd for C_20_H_13_ClN_4_O: 360.372, found: 361.0514.

**4-(4-Bromophenyl)-3-(4-chlorophenyl)-6-oxo-6,7-dihydro-1*H*-pyrazolo[3,4-*b*]pyridine-5-carbonitrile (9a).**

White solid; M.p: >300 ˚C; (*n-*Hexane: Ethyl acetate 2:8). FT-IR (KBr, cm^-1^): 3176, 2241, 1652, 1603, 1532. ^1^H NMR (400 MHz, DMSO*-d_6_*) δ_ppm_ 13.40 (s, 2H), 7.42 (d, *J* = 8.5 Hz, 2H), 7.24 – 7.13 (m, 4H), 6.94 (d, *J* = 8.6 Hz, 2H). ^13^C NMR (101 MHz, DMSO*-d_6_*) δ_ppm_ 164.8, 154.2, 150.2, 142.2, 133.2, 133.0, 130.8, 130.4, 130.4, 127.5, 122.9, 116.8, 116.8, 102.9. Mass spectrum calcd for C_19_H_10_BrClN_4_O: 423.9727, found: 424.9925.

**3,4-Bis(4-chlorophenyl)-6-oxo-6,7-dihydro-1*H*-pyrazolo[3,4-*b*]pyridine-5-carbonitrile (10a).**

White solid; M.p: >300 ˚C; (*n-*Hexane: Ethyl acetate 2:8). FT-IR (KBr, cm^-1^): 3180, 2241, 1651, 1595. ^1^H NMR (400 MHz, DMSO*-d_6_*) δ_ppm_ 14.06 (s, 1H), 12.82 (s, 1H), 7.40 (d, *J* = 8.6 Hz, 2H), 7.35 (d, *J* = 6.4 Hz, 2H), 7.30 (d, *J* = 8.5 Hz, 2H), 7.06 (d, *J* = 8.6 Hz, 2H). ^13^C NMR (101 MHz, DMSO*-d_6_*) δ_ppm_ 168.4, 155.6, 148.5, 147.1, 146.0, 143.6, 142.4, 132.1, 131.9, 130.2, 130.0, 127.1, 122.7, 119.3, 101.2. Mass spectrum calcd for C_19_H_10_Cl_2_N_4_O: 380.232, found: 381.2864

**3-(4-Chlorophenyl)-6-oxo-4-phenyl-6,7-dihydro-1*H*-pyrazolo[3,4-*b*]pyridine-5-carbonitrile (11a).**

Brown solid; M.p: >300 ˚C; (*n-*Hexane: Ethyl acetate 2:8). FT-IR (KBr, cm^-1^): 3428, 3172, 2925, 2856, 2226, 1652. ^1^H NMR (400 MHz, DMSO*-d_6_*) δ_ppm_ 12.24 (s, 2H), 7.35 (d, *J* = 6.7 Hz, 1H), 7.25 – 7.17 (m, 4H), 7.09 (d, *J* = 8.5 Hz, 2H), 6.91 (d, *J* = 8.5 Hz, 2H). ^13^C NMR (101 MHz, DMSO*-d_6_*) δ_ppm_ 162.9, 155.2, 150.4, 142.4, 134.0, 132.9, 130.3, 129.3, 128.3, 128.0, 127.5, 117.1, 102.8.

**4-(4-Chlorophenyl)-3-(1*H*-indol-3-yl)-6-oxo-1-phenyl-6,7-dihydro-1*H*-pyrazolo[3,4-*b*]pyridine-5-carbonitrile (1b).**

Yellow solid; M.p: >300 ˚C; (*n-*Hexane: Ethyl acetate 7:3). FT-IR (KBr, cm^-1^): 3434, 3216, 2985, 2917, 225, 1633, 1613. ^1^H NMR (400 MHz, DMSO*-d_6_*) δ_ppm_ 13.33 (s, 1H), 11.32 (s, 1H), 8.36 (d, *J* = 8.1 Hz, 2H), 8.09 (d, *J* = 7.7 Hz, 1H), 7.73 (t, *J* = 8.7, Hz, 2H), 7.55 – 7.49 (m, 5H), 7.44 (d, *J* = 8.1 Hz, 1H), 7.23 (t, *J* = 7.5 Hz, 1H), 7.15 (t, *J* = 8.1 Hz, 1H), 6.21 (d, *J* = 2.8 Hz, 1H). ^13^C NMR (101 MHz, DMSO*-d_6_*) δ_ppm_ 163.8, 152.5, 142.2, 138.4, 135.5, 134.6, 133.0, 130.9, 129.2, 128.2, 126.4, 125.7, 121.7, 121.4, 120.6, 119.7, 115.9, 111.4, 108.1, 106.0.

**3-(1*H*-indol-3-yl)-4-(4-isopropylphenyl)-6-oxo-1-phenyl-6,7-dihydro-1*H*-pyrazolo[3,4-*b*]pyridine-5-carbonitrile (2b).**

Yellow solid; M.p: >300 ˚C; (*n-*Hexane: Ethyl acetate 7:3). FT-IR (KBr, cm^-1^): 3413, 2958, 2924, 2856, 2216, 1659, 1535. ^1^H NMR (400 MHz, DMSO*-d_6_*) δ_ppm_ 11.94 (s, 1H), 11.10 (s, 1H), 8.50 (d, *J* = 7.9 Hz, 1H), 8.47 (d, *J* = 3.0 Hz, 1H), 8.42 (d, *J* = 7.5 Hz, 1H), 8.16 (d, *J* = 7.3 Hz, 1H), 7.68 (t, *J* = 7.5 Hz, 2H), 7.57 (d, *J* = 7.4 Hz, 1H), 7.45 (d, *J* = 8.3 Hz, 2H), 7.33 (t, *J* = 8.4 Hz, 1H), 7.25 (t, *J* = 5.1 Hz, 1H), 7.14 (t, *J* = 7.2 Hz, 1H), 7.09 (t, *J* = 5.6 Hz, 1H), 5.86 (d, *J* = 2.7 Hz, 1H), 2.99 (h, *J* = 6.9 Hz, 1H), 1.30 (d, *J* = 6.9 Hz, 6H). ^13^C NMR (101 MHz, DMSO*-d_6_*) δ_ppm_ 164.2, 157.5, 153.0, 150.4, 146.4, 139.4, 136.3, 132.4, 129.2, 126.2, 124.0, 122.6, 121.5, 119.0, 112.2, 106.5, 29.0, 23.8.

**3-(1*H*-indol-3-yl)-6-oxo-1-phenyl-4-(pyridin-4-yl)-6,7-dihydro-1*H*-pyrazolo[3,4-*b*]pyridine-5-carbonitrile (3b).**

Yellow solid; M.p: >300 ˚C; (*n-*Hexane: Ethyl acetate 7:3). FT-IR (KBr, cm^-1^): 3808, 2232, 1638, ^1^H NMR (400 MHz, DMSO*-d_6_*) δ_ppm_ 11.56 – 11.21 (m, 2H), 8.58 (d, *J* = 6.0 Hz, 2H), 8.24 (d, *J* = 7.8 Hz, 2H), 7.92 (d, *J* = 7.8 Hz, 1H), 7.64 (t, *J* = 8.0 Hz, 2H), 7.45 (d, *J* = 6.0 Hz, 2H), 7.32 (d, *J* = 8.2 Hz, 1H), 7.12 (t, *J* = 7.5 Hz, 2H), 7.04 (t, *J* = 6.9 Hz, 1H), 6.19 (d, *J* = 2.8 Hz, 1H). ^13^C NMR (101 MHz, DMSO*-d_6_*) δ_ppm_ 163.6, 150.9, 149.2, 143.4, 142.0, 138.3, 135.5, 129.4, 128.0, 126.7, 125.8, 125.4, 123.7, 121.8, 121.6, 120.9, 120.3, 119.7, 115.4, 113.4, 111.4, 107.9, 105.8. Mass spectrum calcd for C_26_H_16_N_6_O: 428.1386, found: 429.0213.

**3-(1*H*-indol-3-yl)-4-(3-nitrophenyl)-6-oxo-1-phenyl-6,7-dihydro-1*H*-pyrazolo[3,4-*b*]pyridine-5-carbonitrile (4b).**

Yellow solid; M.p: >300 ˚C; (*n*-Hexane: Ethyl acetate 7:3). ^1^H NMR (400 MHz, DMSO*-d_6_*) δ_ppm_ 11.02 (s, 1H), 8.35 (d, *J* = 8.2 Hz, 2H), 8.07 – 8.04 (m, 2H), 7.72 (d, *J* = 7.5 Hz, 1H), 7.60 (d, *J* = 7.8 Hz, 1H), 7.53 (t, *J* = 6.4 Hz, 2H), 7.45 (t, *J* = 7.2 Hz, 1H), 7.28 (t, *J* = 7.4 Hz, 1H), 7.22 (d, *J* = 8.2 Hz, 1H), 7.00 (t, *J* = 6.9 Hz, 1H), 6.87 (t, *J* = 6.9 Hz, 1H), 6.40 (d, *J* = 2.7 Hz, 1H). ^13^C NMR (101 MHz, DMSO*-d_6_*) δ_ppm_ 169.0, 149.9, 149.1, 146.4, 141.2, 139.6, 136.3, 135.5, 135.3, 129.1, 128.8, 126.0, 125.8, 123.7, 123.3, 121.3, 120.2, 119.9, 119.1, 111.0, 106.9. Mass spectrum calcd for C_27_H_16_N_6_O_3_: 472.1284, found: 473.1087

**3-(1*H*-indol-3-yl)-6-oxo-1-phenyl-4-(*m*-tolyl)-6,7-dihydro-1*H*-pyrazolo[3,4-*b*]pyridine-5-carbonitrile (5b).**

Yellow solid; M.p: >300 ˚C; (*n*-Hexane: Ethyl acetate 7:3). FT-IR (KBr, cm^-1^): 3414, 3264, 3057, 2221, 1629. ^1^H NMR (400 MHz, DMSO*-d_6_*) δ_ppm_ 11.37 (s, 1H), 11.06 (s, 1H), 8.34 (d, *J* = 8.6 Hz, 2H), 8.06 (d, *J* = 7.7 Hz, 1H), 7.59 (t, *J* = 8.0 Hz, 1H), 7.53 (t, *J* = 7.7 Hz, 2H), 7.29 – 7.27 (m, 3H), 7.17 (d, *J* = 6.7 Hz, 1H), 7.10 (d, *J* = 5.4 Hz, 2H), 7.07 (d, *J* = 5.2 Hz, 1H), 5.83 (d, *J* = 2.7 Hz, 1H), 2.15 (s, 3H). ^13^C NMR (101 MHz, DMSO*-d_6_*) δ_ppm_ 163.3, 152.7, 142.0, 139.3, 137.3, 135.5, 129.7, 129.4, 129.3, 128.0, 126.1, 125.9, 125.8, 125.2, 122.6, 121.5, 120.5, 119.5, 111.1, 106.7, 20.3.

**4-(2,4-Difluorophenyl)-3-(1*H*-indol-3-yl)-6-oxo-1-phenyl-6,7-dihydro-1*H*-pyrazolo[3,4-*b*]pyridine-5-carbonitrile (6b).**

Brown solid; M.p: >300 ˚C; (*n*-Hexane: Ethyl acetate 7:3). FT-IR (KBr, cm^-1^): 3428, 3172, 2925, 2856, 2226, 1652. ^1^H NMR (400 MHz, DMSO*-d_6_*) δ_ppm_ 12.00 (s, 1H), 11.32 (s, 1H), 8.48 (d, *J* = 2.6 Hz, 1H), 8.41 (d, *J* = 8.7 Hz, 2H), 7.96 (d, *J* = 7.9 Hz, 1H), 7.70 (d, *J* = 6.8 Hz, 1H), 7.58 (d, *J* = 7.8 Hz, 1H), 7.47 (d, *J* = 1.6 Hz, 1H), 7.39 (d, *J* = 7.0 Hz, 1H), 7.29 (t, *J* = 5.6 Hz, 1H), 7.23 (t, *J* = 5.4 Hz, 1H), 7.14 (t, *J* = 8.1 Hz, 1H), 7.08 (t, *J* = 6.9 Hz, 1H), 6.33 (d, *J* = 2.8 Hz, 1H). ^13^C NMR (101 MHz, DMSO*-d_6_*) δ_ppm_ 150.1, 129.3, 126.7, 125.8, 122.6, 121.5, 120.4, 119.7, 112.2, 111.4.

**4-(4-Bromophenyl)-3-(1*H*-indol-3-yl)-6-oxo-1-phenyl-6,7-dihydro-1*H*-pyrazolo[3,4-*b*]pyridine-5-carbonitrile (7b).**

Yellow solid; M.p: >300 ˚C; (*n*-Hexane: Ethyl acetate 7:3). FT-IR (KBr, cm^-1^): 3419, 2923, 2852, 2216, 1609, 1573. ^1^H NMR (400 MHz, DMSO*-d_6_*) δ_ppm_ 11.99 (s, 1H), 11.31 (s, 1H), 8.50 (d, *J* = 7.3 Hz, 1H), 8.47 (d, *J* = 2.7 Hz, 1H), 8.42 (d, *J* = 7.4 Hz, 2H), 8.09 (d, *J* = 7.7 Hz, 1H), 7.69 (t, *J* = 7.5 Hz, 1H), 7.64 (d, *J* = 8.5 Hz, 2H), 7.50 (d, *J* = 8.3 Hz, 2H), 7.37 (d, *J* = 7.9 Hz, 1H), 7.28 (t, *J* = 5.9 Hz, 1H), 7.15 (t, *J* = 5.9 Hz, 1H), 6.14 (d, *J* = 2.7 Hz, 1H). ^13^C NMR (101 MHz, DMSO*-d_6_*) δ_ppm_ 164.5, 155.3, 151.6, 142.1, 138.5, 136.4, 135.6, 133.8, 131.4, 131.2, 129.5, 129.2, 126.5, 125.8, 123.4, 122.6, 121.8, 121.5, 120.9, 119.9, 118.8, 112.6, 112.2, 111.5, 110.2, 106.0, 99.5.

**D_2_O exchange spectrum of 4-(4-bromophenyl)-3-(1*H*-indol-3-yl)-6-oxo-1-phenyl-6,7-dihydro-1*H*-pyrazolo[3,4-*b*]pyridine-5-carbonitrile (7b).**

^1^H NMR (400 MHz, D_2_O*-d_6_*) δ_ppm_ 8.20 (d, *J* = 8.0 Hz, 1H), 8.17 (s, 1H), 8.08 (d, *J* = 8.0 Hz, 2H), 7.73 (d, *J* = 7.9 Hz, 1H), 7.39 (t, *J* = 7.8 Hz, 2H), 7.29 (d, *J* = 8.8 Hz, 2H), 7.16 (d, *J* = 8.4 Hz, 2H), 7.04 (t, *J* = 7.5 Hz, 1H), 6.90 (t, *J* = 7.5 Hz, 1H), 5.93 (s, 1H).

**4,4'-(1,4-Phenylene)bis(3-(1*H*-indol-3-yl)-6-oxo-1-phenyl-6,7-dihydro-1*H*-pyrazolo[3,4-*b*]pyridine-5-carbonitrile (8b).**

Yellow solid; M.p: >300 ˚C; (*n*-Hexane: Ethyl acetate 7:3). FT-IR (KBr, cm^-1^): 3422, 2928, 2856, 2216, 1741, 1653. ^1^H NMR (400 MHz, DMSO*-d_6_*) δ_ppm_ 10.10 (m, 4H), 8.35 – 8.31 (m, 4H), 8.25 – 8.21 (m, 2H), 7.63 – 7.61 (m, 4H), 7.42 – 7.39 (m, 4H), 7.22 – 7.19 (m, 2H), 7.10 – 7.06 (m, 6H), 5.95 (s, 2H). Mass spectrum calcd for C_48_H_28_N_10_O: 776.2397, found: 777.125.

**4,4'-(1,4-Phenylene)bis(3-(4-chlorophenyl)-6-oxo-6,7-dihydro-1*H*-pyrazolo[3,4-*b*]pyridine-5-carbonitrile) (12a).**

Yellow solid; M.p: >300 ˚C; (*n*-Hexane: Ethyl acetate 2:8). FT-IR (KBr, cm^-1^): 3423, 3258, 2942, 2859, 2213, 1653. ^1^H NMR (400 MHz, DMSO*-d_6_*) δ_ppm_ 13.03 (m, 4H), 8.03 – 7.93 (m, 2H), 7.54 – 7.46 (m, 4H), 7.21 – 7.14 (m, 4H), 6.86 – 6.80 (m, 2H). Mass spectrum calcd for C_32_H_16_Cl_2_N_8_O: 380.232, found: 381.2769.


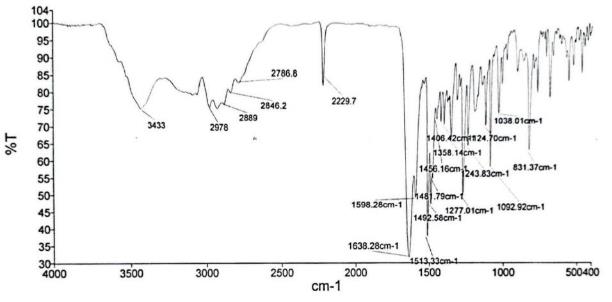


# FT-IR spectrum of 3-(4-chlorophenyl)-4-(3-ethoxy-4-hydroxyphenyl)-6-oxo-6,7-dihydro-1*H*-pyrazolo[3,4-*b*]pyridine-5-carbonitrile (1a).


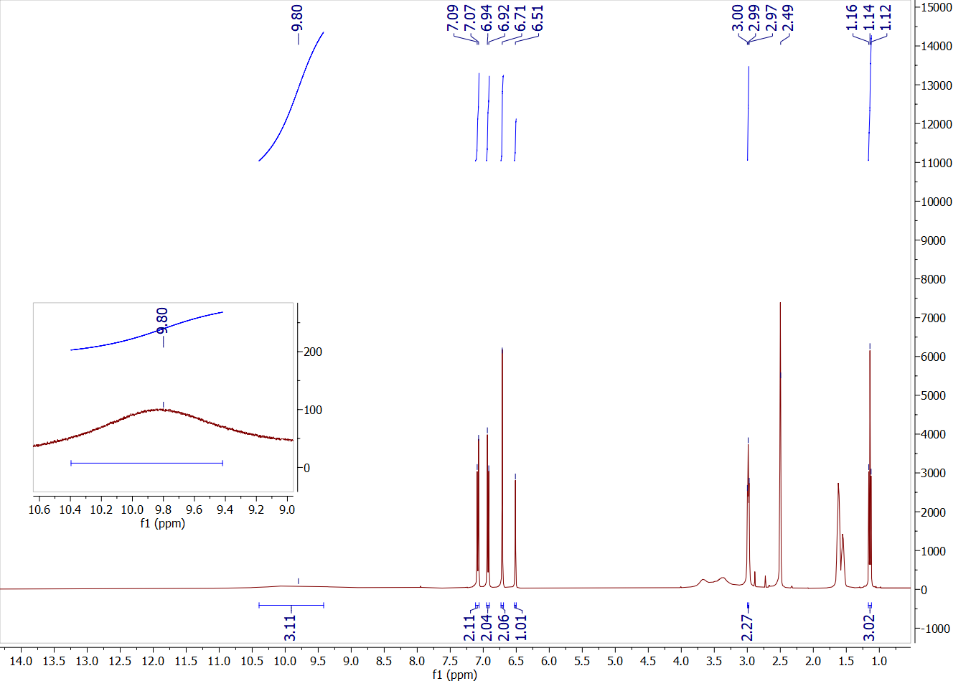


# ^1^H-NMR spectrum of 3-(4-chlorophenyl)-4-(3-ethoxy-4-hydroxyphenyl)-6-oxo-6,7-dihydro-1*H*-pyrazolo[3,4-*b*]pyridine-5-carbonitrile (1a).


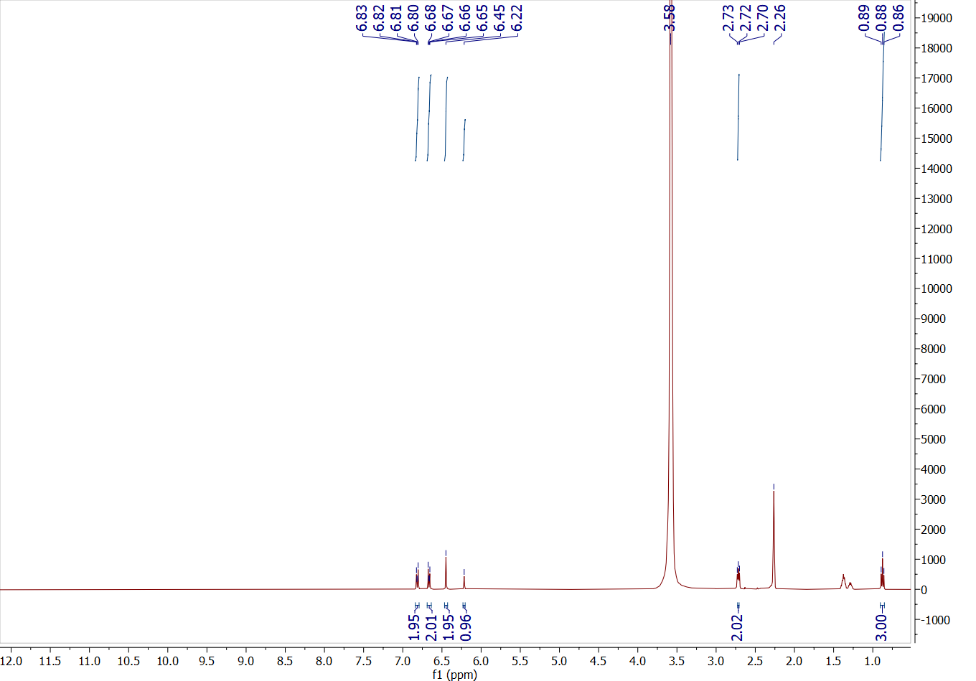


# D_2_O exchange spectrum of 3-(4-chlorophenyl)-4-(3-ethoxy-4-hydroxyphenyl)-6-oxo-6,7-dihydro-1*H*-pyrazolo[3,4-*b*]pyridine-5-carbonitrile (1a).


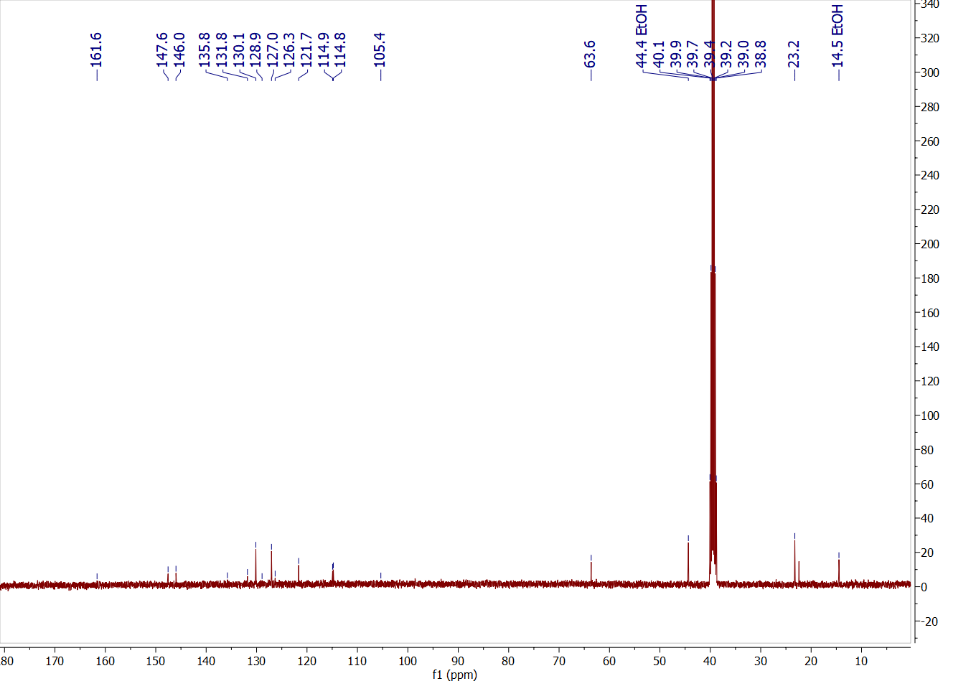


# ^13^C-NMR spectrum of 3-(4-chlorophenyl)-4-(3-ethoxy-4-hydroxyphenyl)-6-oxo-6,7-dihydro-1*H*-pyrazolo[3,4-*b*]pyridine-5-carbonitrile (1a).


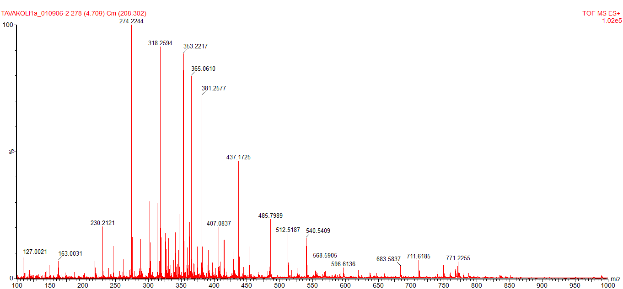


# Mass spectrum of 3-(4-chlorophenyl)-4-(3-ethoxy-4-hydroxyphenyl)-6-oxo-6,7-dihydro-1*H*-pyrazolo[3,4-*b*]pyridine-5-carbonitrile (1a).


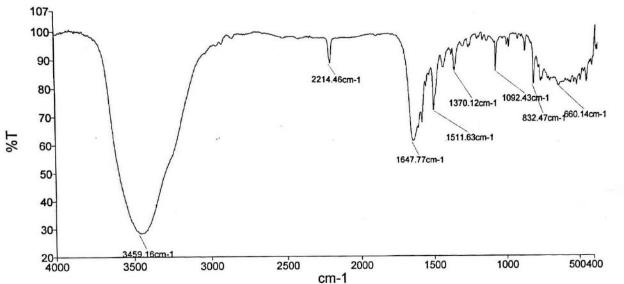


# FT-IR spectrum of 3-(4-chlorophenyl)-4-(4-methoxyphenyl)-6-oxo-6,7-dihydro-1*H*-pyrazolo[3,4-*b*]pyridine-5-carbonitrile (2a).


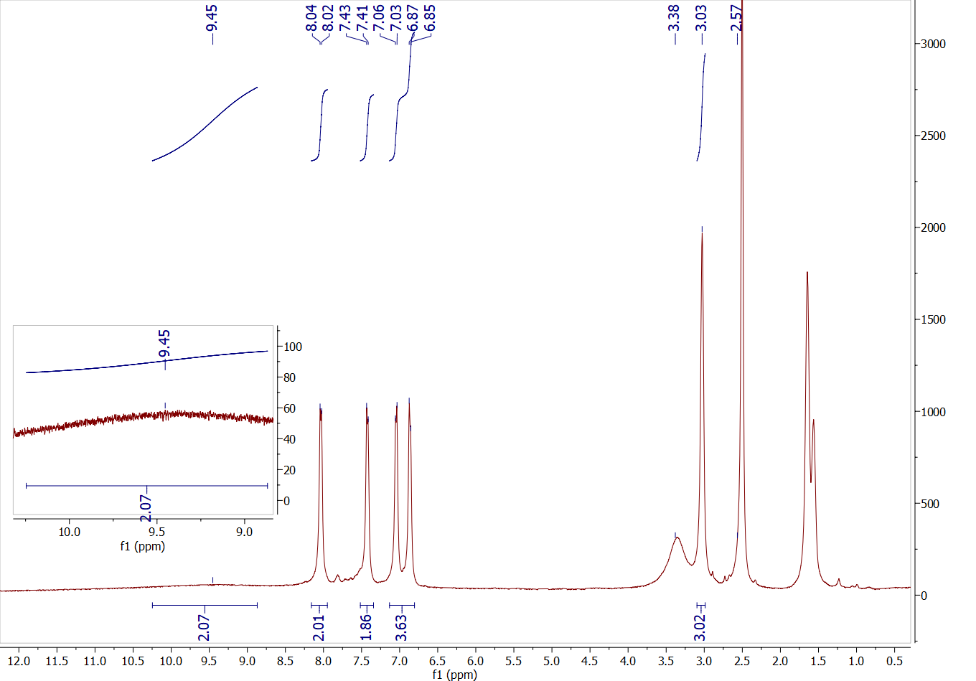


# ^1^H-NMR spectrum of 3-(4-chlorophenyl)-4-(4-methoxyphenyl)-6-oxo-6,7-dihydro-1*H*-pyrazolo[3,4-*b*]pyridine-5-carbonitrile (2a).


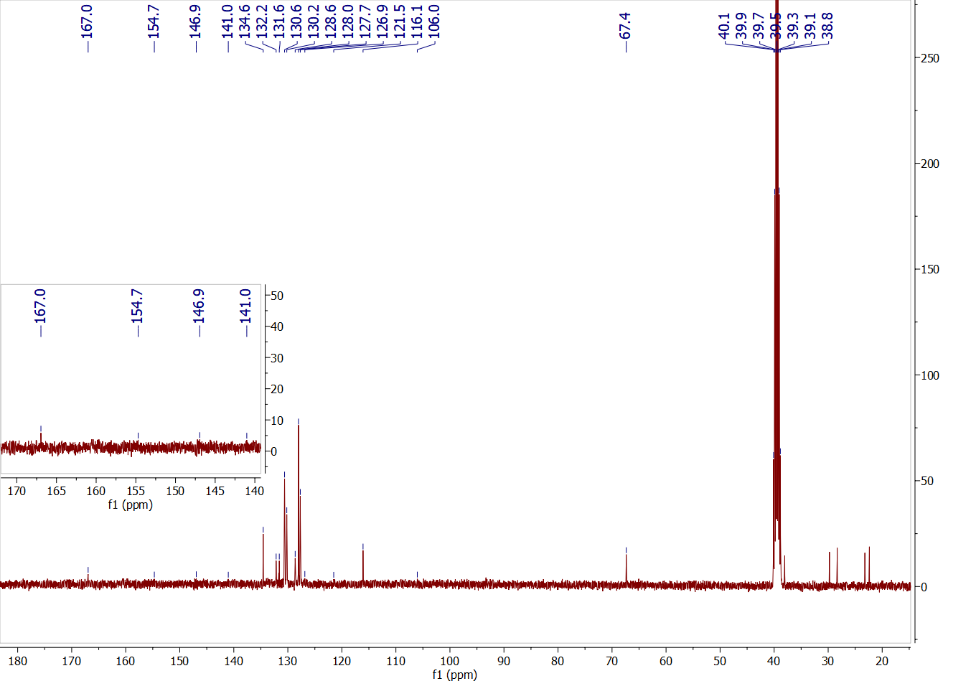


# ^13^C-NMR spectrum of 3-(4-chlorophenyl)-4-(4-methoxyphenyl)-6-oxo-6,7-dihydro-1*H*-pyrazolo[3,4-*b*]pyridine-5-carbonitrile (2a).


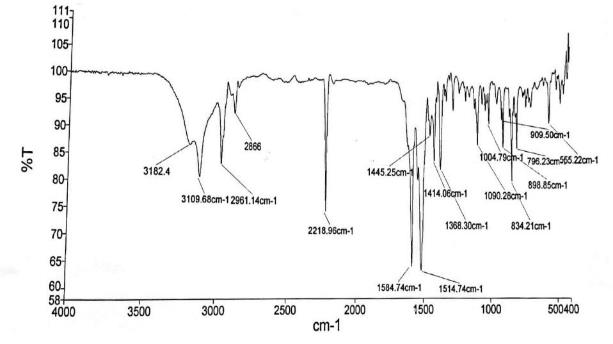


# FT-IR spectrum of 3-(4-chlorophenyl)-4-(4-isopropylphenyl)-6-oxo-6,7-dihydro-1*H*-pyrazolo[3,4-*b*]pyridine-5-carbonitrile (3a).


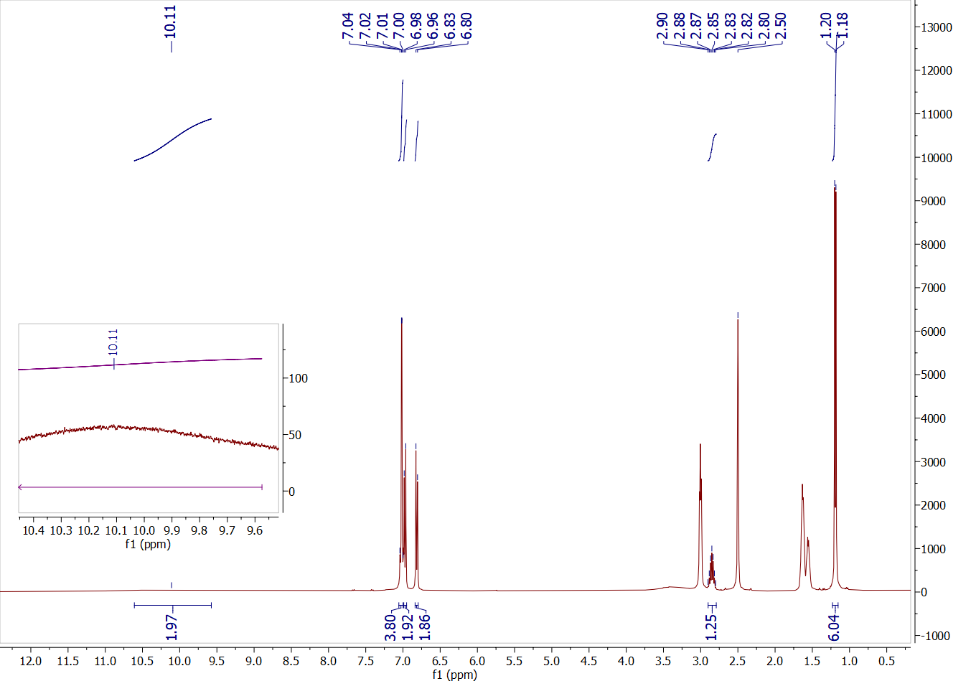


# ^1^H-NMR spectrum of 3-(4-chlorophenyl)-4-(4-isopropylphenyl)-6-oxo-6,7-dihydro-1*H*-pyrazolo[3,4-*b*]pyridine-5-carbonitrile (3a).


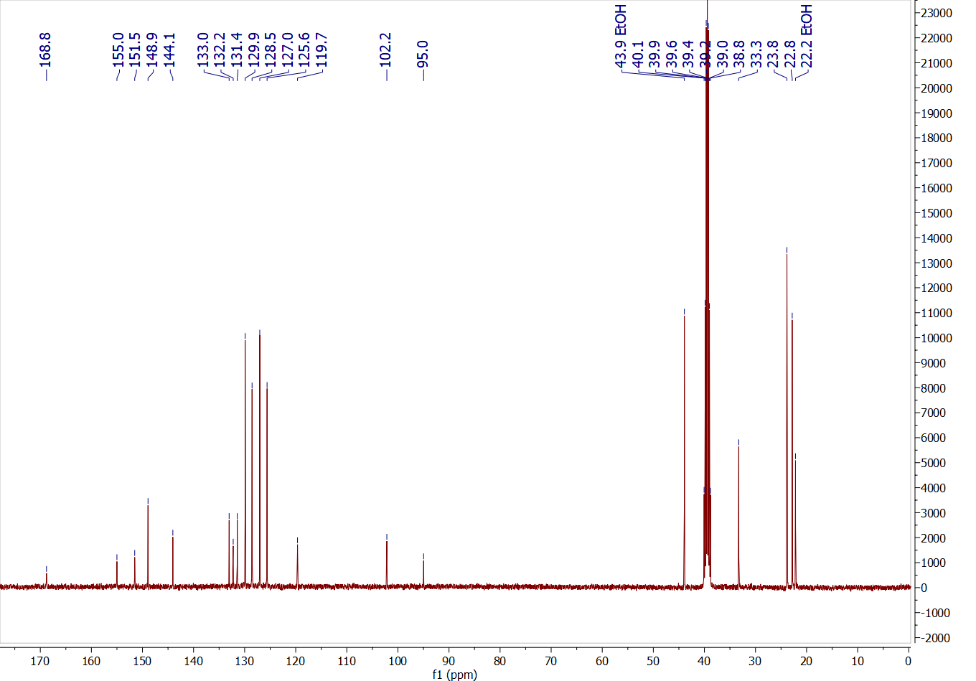


# ^13^C-NMR spectrum of 3-(4-chlorophenyl)-4-(4-isopropylphenyl)-6-oxo-6,7-dihydro-1*H*-pyrazolo[3,4-*b*]pyridine-5-carbonitrile (3a).


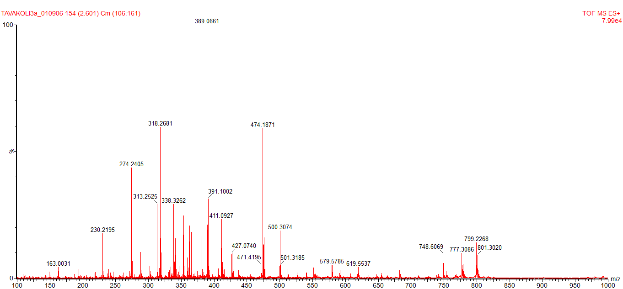


# Mass spectrum of 3-(4-chlorophenyl)-4-(4-isopropylphenyl)-6-oxo-6,7-dihydro-1*H*-pyrazolo[3,4-*b*]pyridine-5-carbonitrile (3a).


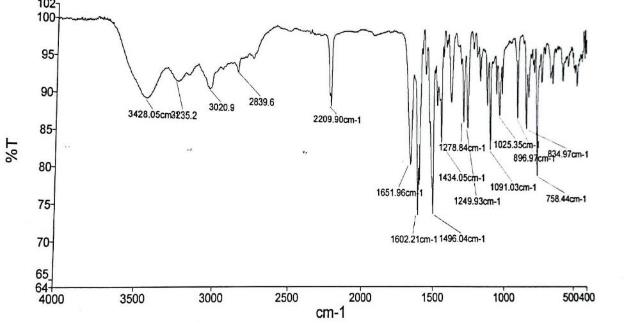


# FT-IR spectrum of 3-(4-chlorophenyl)-4-(2-methoxyphenyl)-6-oxo-6,7-dihydro-1*H*-pyrazolo[3,4-*b*]pyridine-5-carbonitrile (4a).


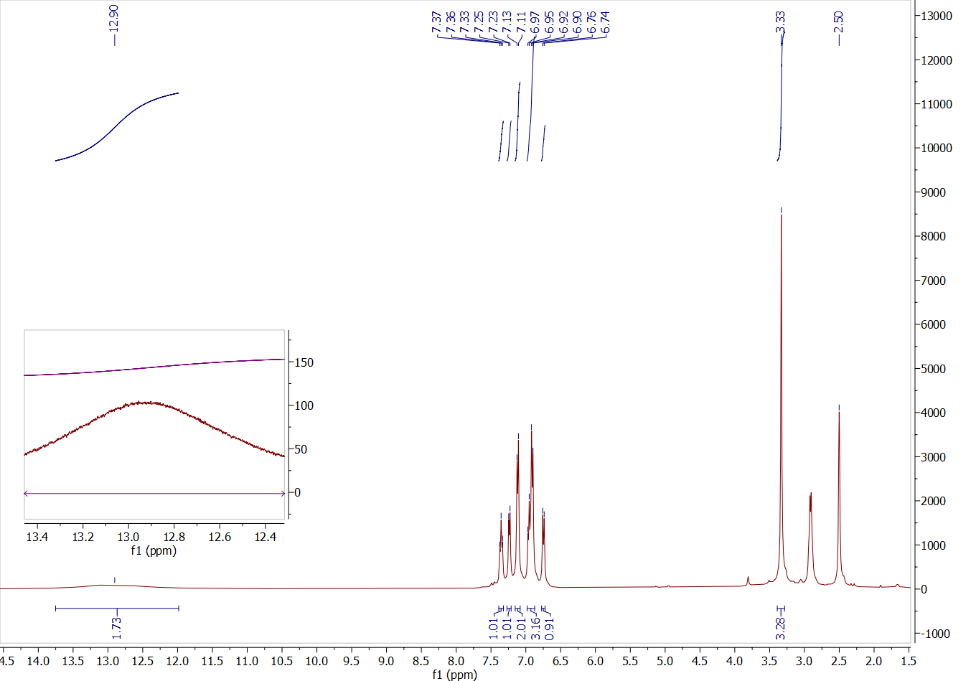


# ^1^H-NMR spectrum of 3-(4-chlorophenyl)-4-(2-methoxyphenyl)-6-oxo-6,7-dihydro-1*H*-pyrazolo[3,4*-b*]pyridine-5-carbonitrile (4a).


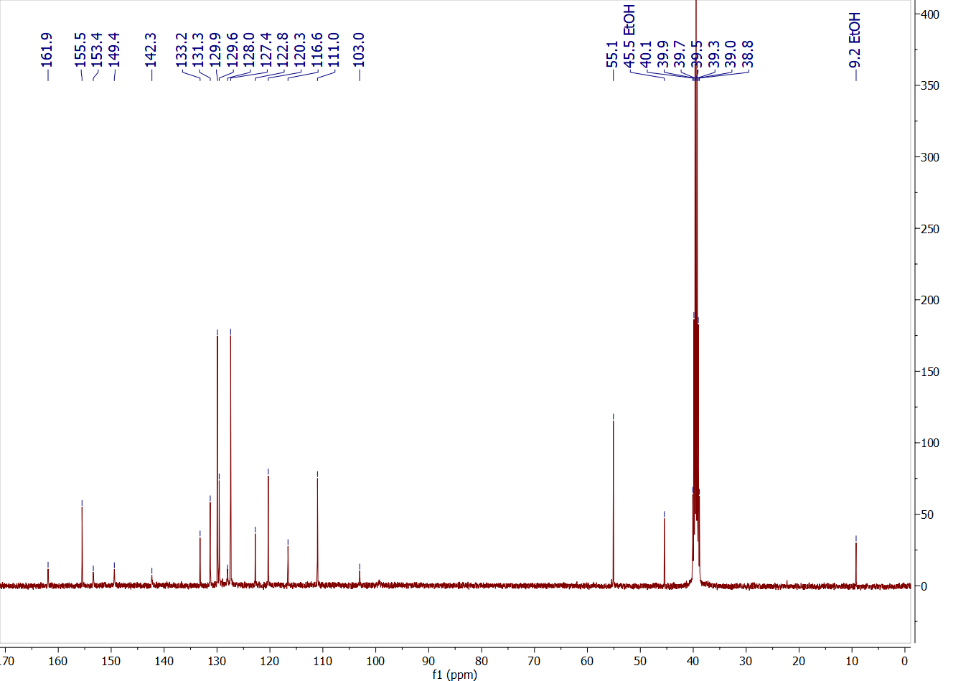


# ^13^C-NMR spectrum of 3-(4-chlorophenyl)-4-(2-methoxyphenyl)-6-oxo-6,7-dihydro-1*H*-pyrazolo[3,4-*b*]pyridine-5-carbonitrile (4a).


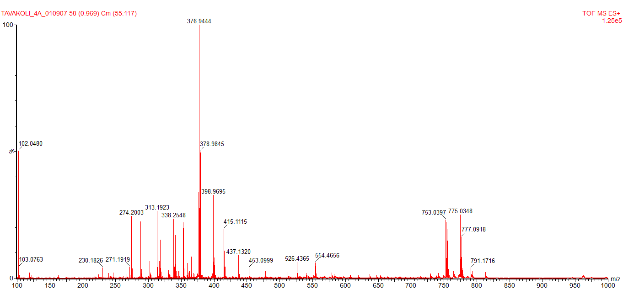


# Mass spectrum of 3-(4-chlorophenyl)-4-(2-methoxyphenyl)-6-oxo-6,7-dihydro-1*H*-pyrazolo[3,4-*b*]pyridine-5-carbonitrile (4a).


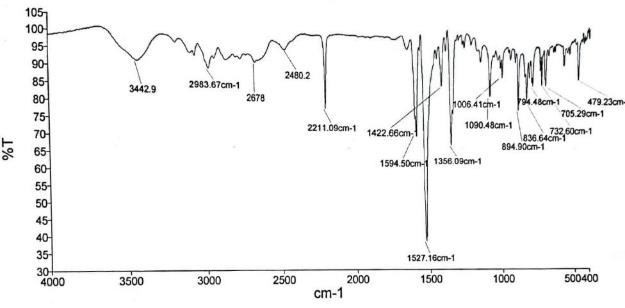


# FT-IR spectrum of 3-(4-chlorophenyl)-4-(3-nitrophenyl)-6-oxo-6,7-dihydro-1*H*-pyrazolo[3,4-*b*]pyridine-5-carbonitrile (5a).


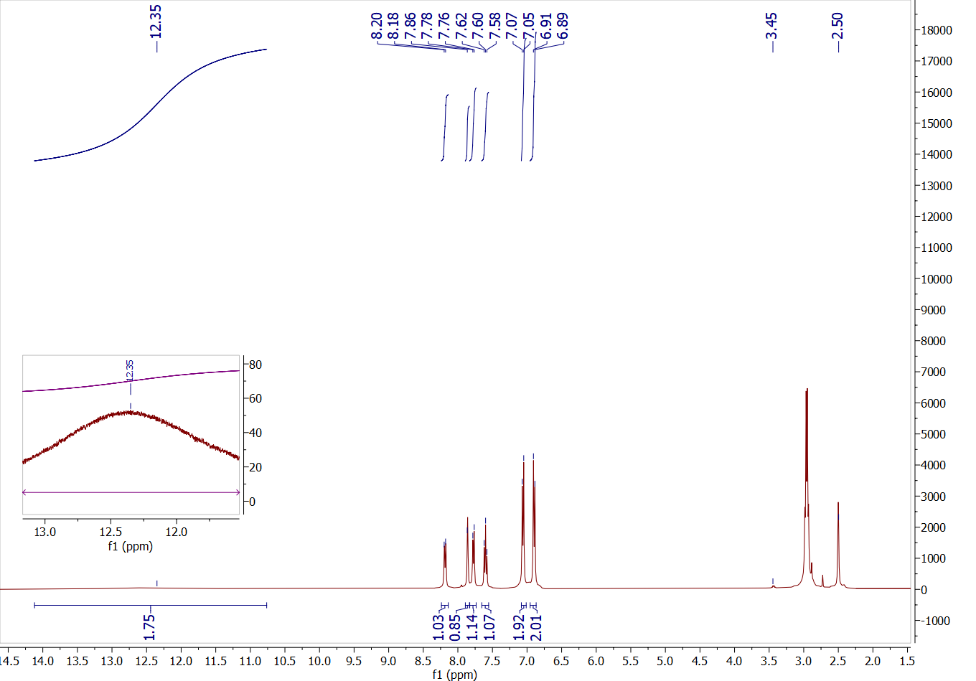


# ^1^H-NMR spectrum of 3-(4-chlorophenyl)-4-(3-nitrophenyl)-6-oxo-6,7-dihydro-1*H*-pyrazolo[3,4-*b*]pyridine-5-carbonitrile (5a).


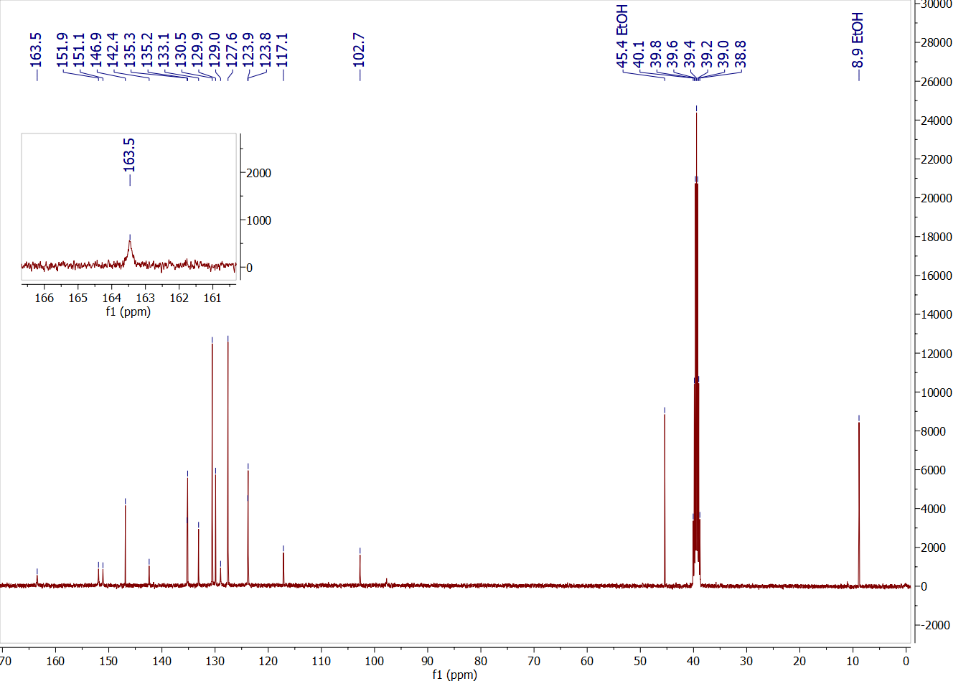


# ^13^C-NMR spectrum of 3-(4-chlorophenyl)-4-(3-nitrophenyl)-6-oxo-6,7-dihydro-1*H*-pyrazolo[3,4-*b*]pyridine-5-carbonitrile (5a).


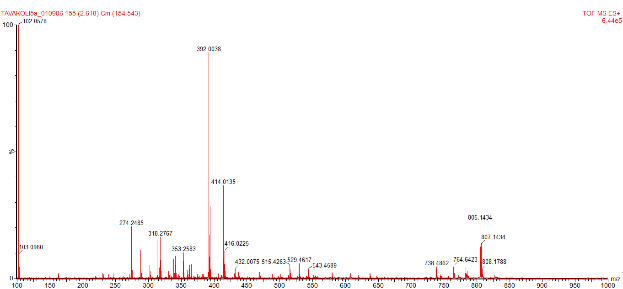


# Mass spectrum of 3-(4-chlorophenyl)-4-(3-nitrophenyl)-6-oxo-6,7-dihydro-1*H*-pyrazolo[3,4-*b*]pyridine-5-carbonitrile (5a).


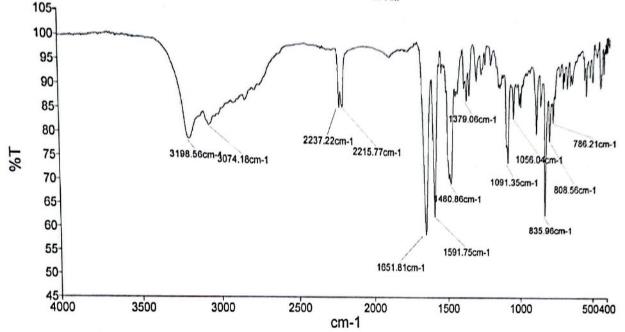


# FT-IR spectrum of 3-(4-chlorophenyl)-4-(2,4-dichlorophenyl)-6-oxo-6,7-dihydro-1*H*-pyrazolo[3,4-*b*]pyridine-5-carbonitrile (6a).


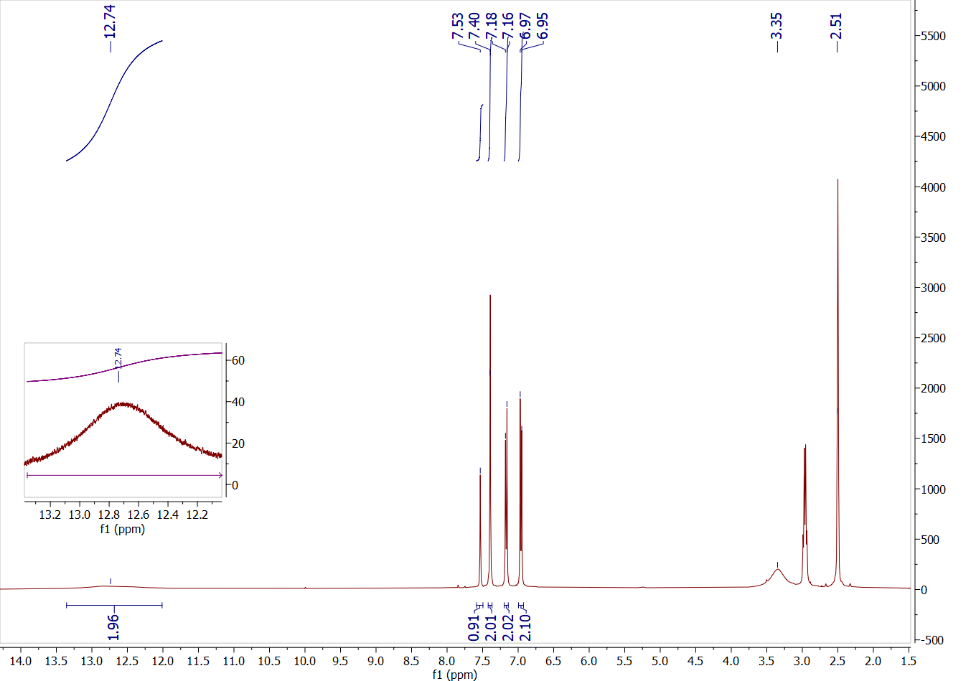


# ^1^H-NMR spectrum of 3-(4-chlorophenyl)-4-(2,4-dichlorophenyl)-6-oxo-6,7-dihydro-1*H*-pyrazolo[3,4-*b*]pyridine-5-carbonitrile (6a).


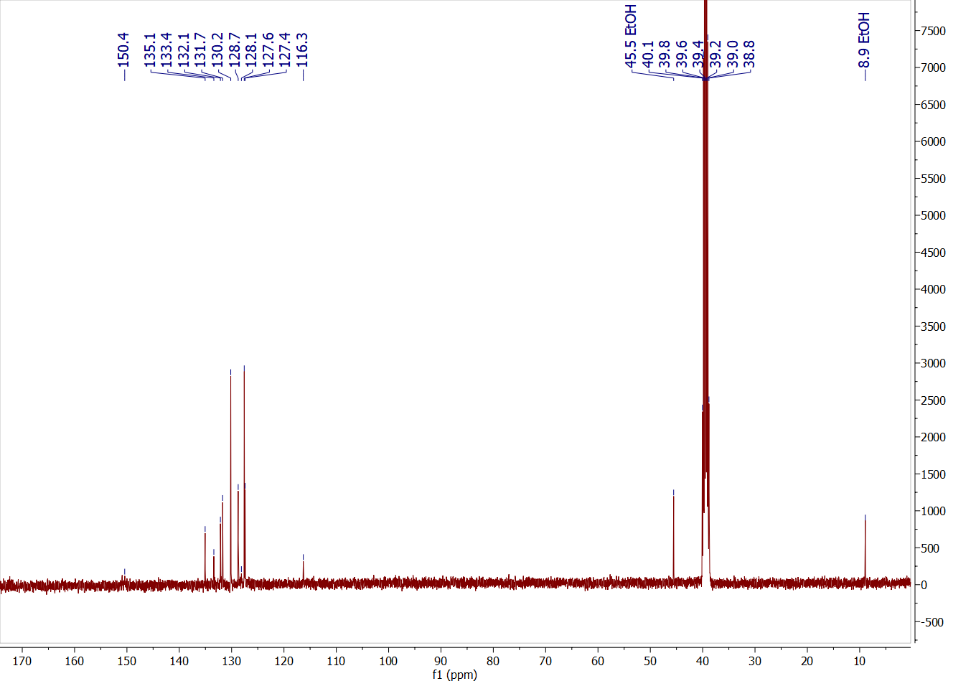


# ^13^C-NMR spectrum of 3-(4-chlorophenyl)-4-(2,4-dichlorophenyl)-6-oxo-6,7-dihydro-1*H*-pyrazolo[3,4-*b*]pyridine-5-carbonitrile (6a).


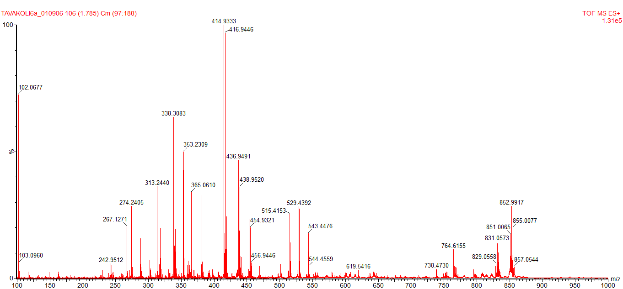


# Mass spectrum of 3-(4-chlorophenyl)-4-(2,4-dichlorophenyl)-6-oxo-6,7-dihydro-1*H*-pyrazolo[3,4-*b*]pyridine-5-carbonitrile (6a).


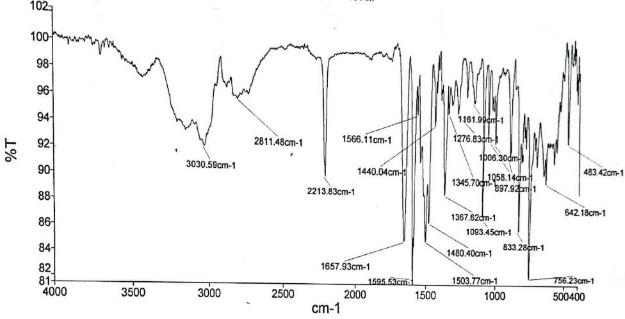


# FT-IR spectrum of 4-(2-chlorophenyl)-3-(4-chlorophenyl)-6-oxo-6,7-dihydro-1*H*-pyrazolo[3,4-*b*]pyridine-5-carbonitrile (7a).


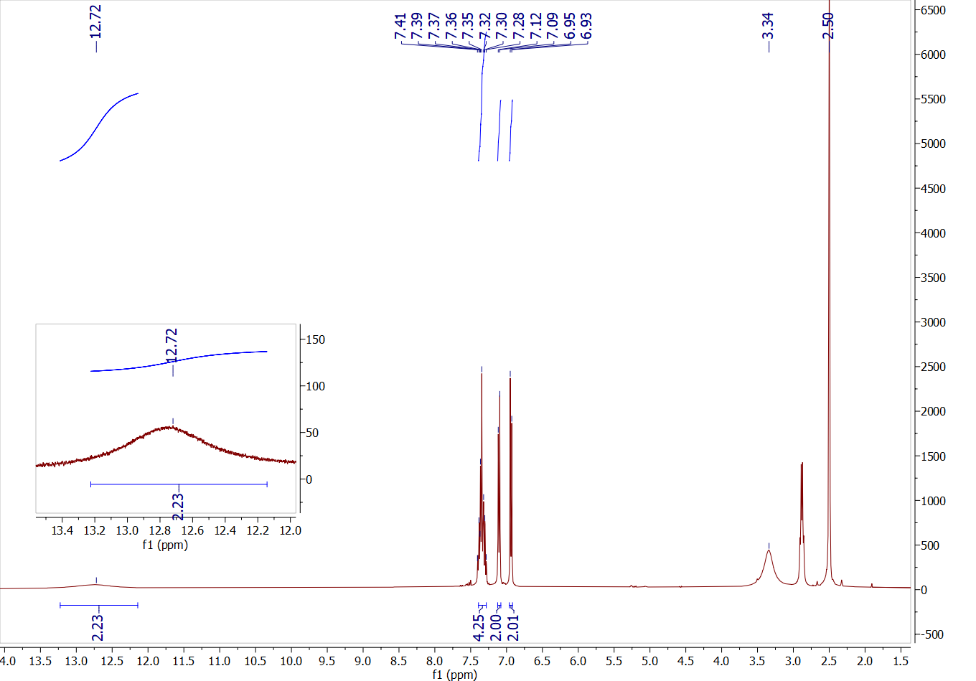


# ^1^H-NMR spectrum of 4-(2-chlorophenyl)-3-(4-chlorophenyl)-6-oxo-6,7-dihydro-1*H*-pyrazolo[3,4-*b*]pyridine-5-carbonitrile (7a).


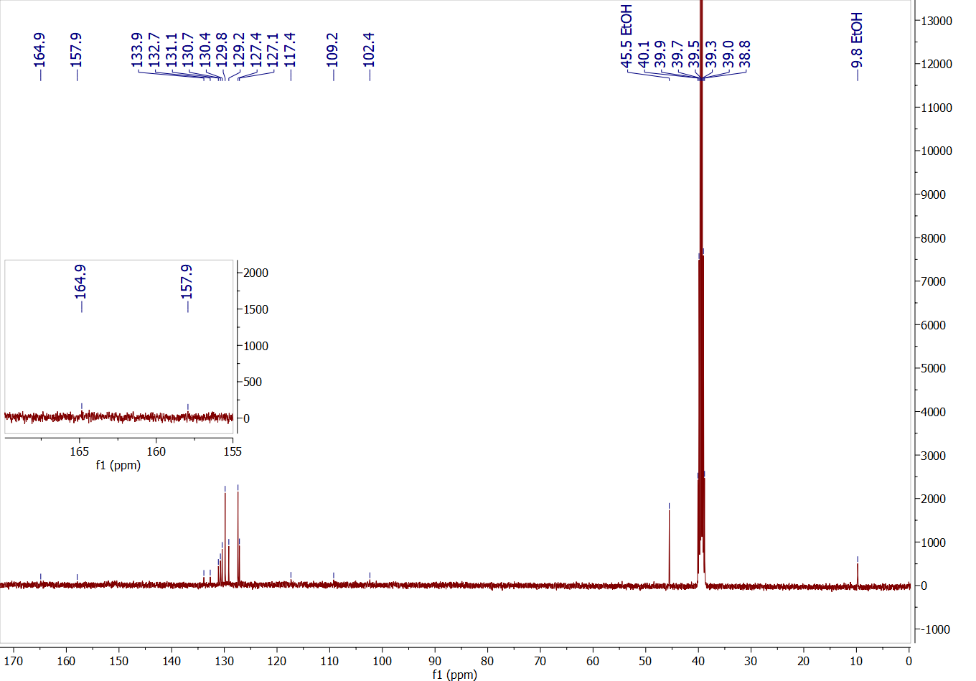


# ^13^C-NMR spectrum of 4-(2-chlorophenyl)-3-(4-chlorophenyl)-6-oxo-6,7-dihydro-1*H*-pyrazolo[3,4-*b*]pyridine-5-carbonitrile (7a).


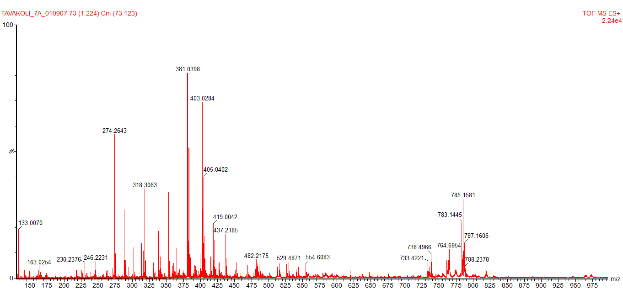


# Mass spectrum of 4-(2-chlorophenyl)-3-(4-chlorophenyl)-6-oxo-6,7-dihydro-1*H*-pyrazolo[3,4-*b*]pyridine-5-carbonitrile (7a).


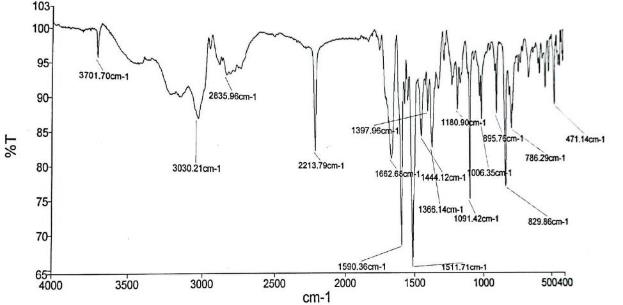


# FT-IR spectrum of 3-(4-chlorophenyl)-6-oxo-4-(*p*-tolyl)-6,7-dihydro-1*H*-pyrazolo[3,4-*b*]pyridine-5-carbonitrile (8a).


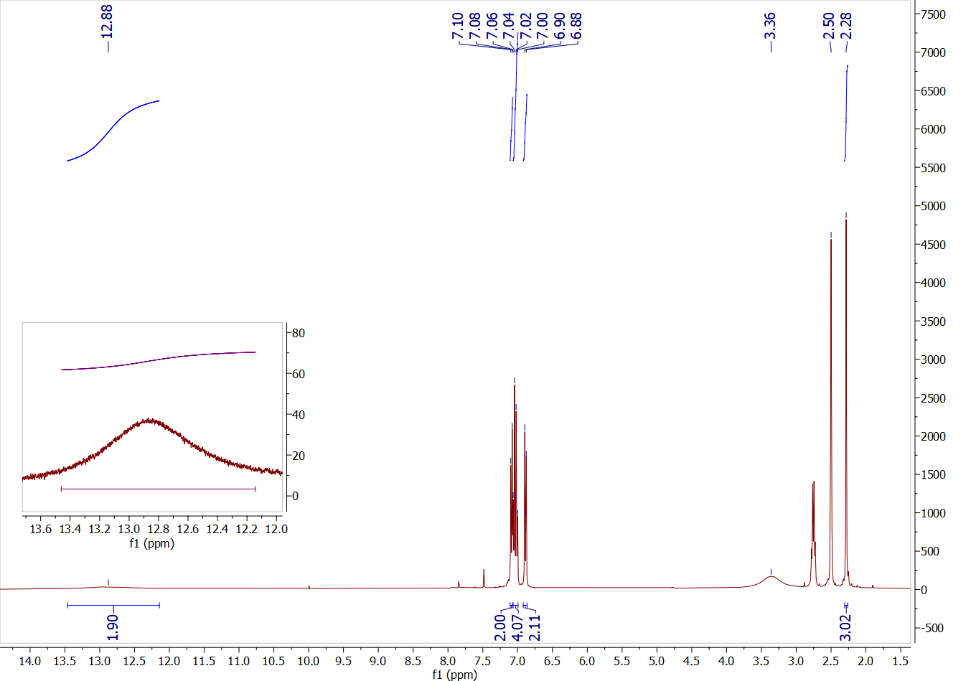


# ^1^H-NMR spectrum of 3-(4-chlorophenyl)-6-oxo-4-(*p*-tolyl)-6,7-dihydro-1*H*-pyrazolo[3,4-*b*]pyridine-5-carbonitrile (8a).


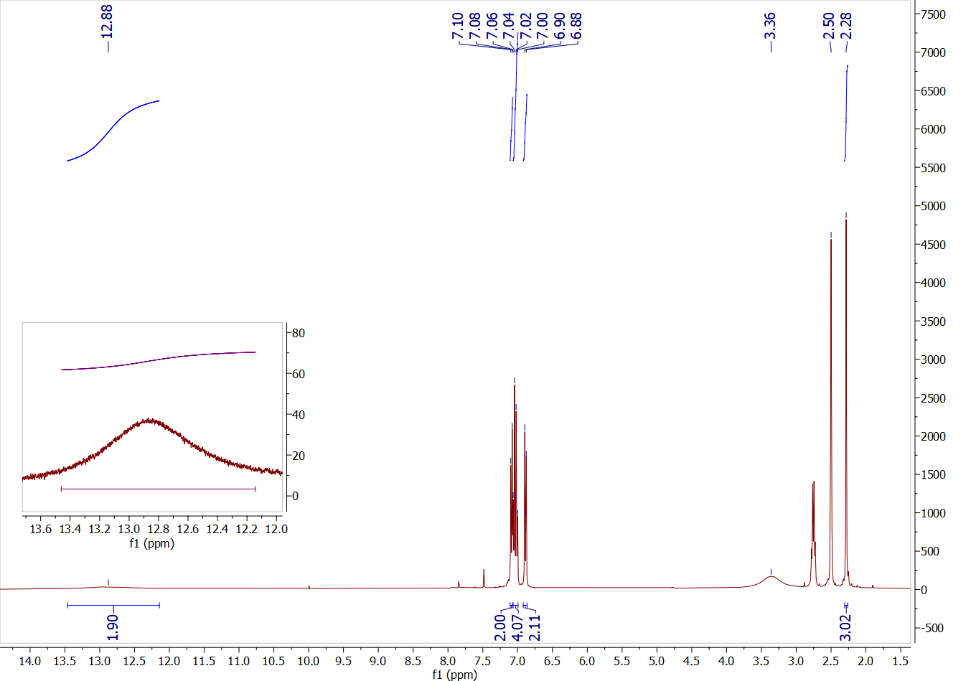


# ^13^C-NMR spectrum of 3-(4-chlorophenyl)-6-oxo-4-(*p*-tolyl)-6,7-dihydro-1*H*-pyrazolo[3,4-b]pyridine-5-carbonitrile (8a).


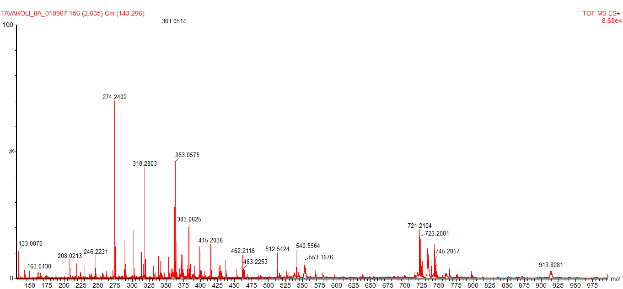


# Mass spectrum of 3-(4-chlorophenyl)-6-oxo-4-(*p*-tolyl)-6,7-dihydro-1*H*-pyrazolo[3,4-b]pyridine-5-carbonitrile (8a).


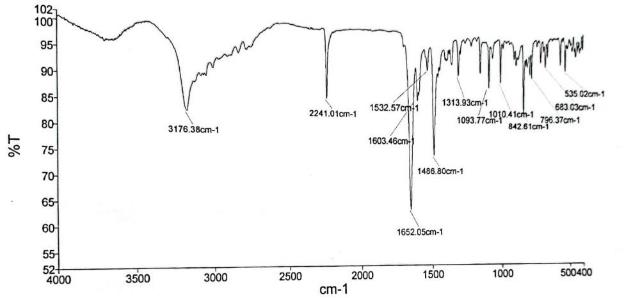


# FT-IR spectrum of 4-(4-bromophenyl)-3-(4-chlorophenyl)-6-oxo-6,7-dihydro-1*H*-pyrazolo[3,4-*b*]pyridine-5-carbonitrile (9a).


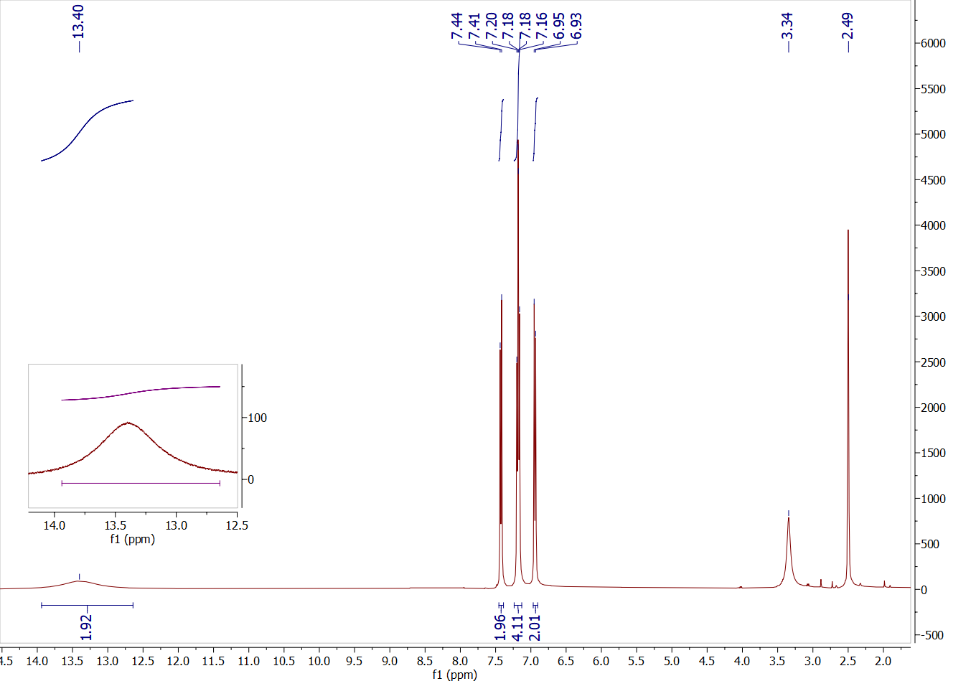


# ^1^H-NMR spectrum of 4-(4-bromophenyl)-3-(4-chlorophenyl)-6-oxo-6,7-dihydro-1*H*-pyrazolo[3,4-*b*]pyridine-5-carbonitrile (9a).


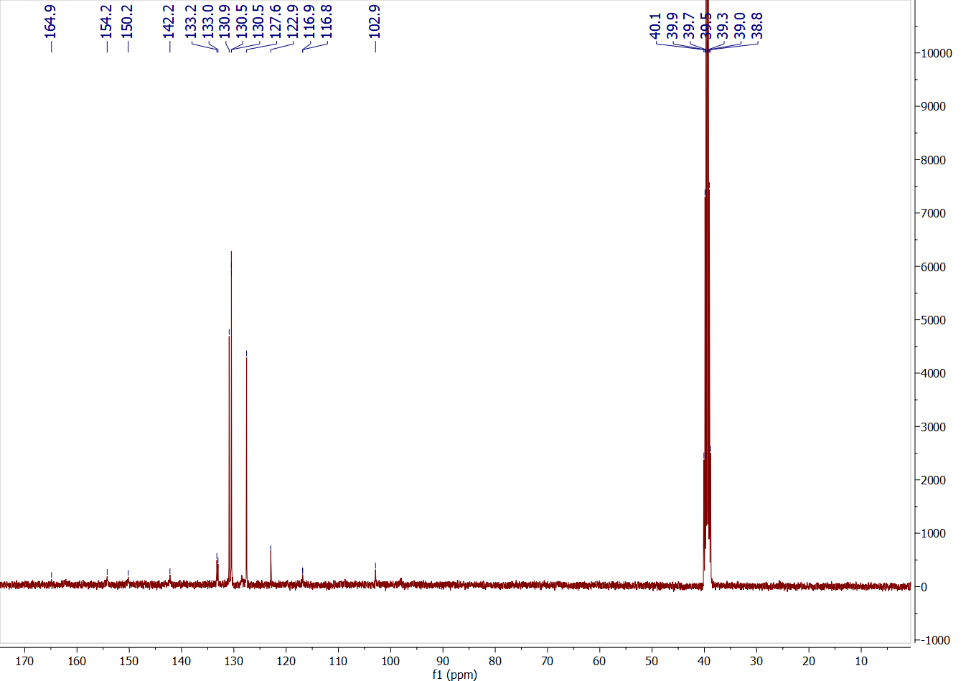


# ^13^C-NMR spectrum of 4-(4-bromophenyl)-3-(4-chlorophenyl)-6-oxo-6,7-dihydro-1*H*-pyrazolo[3,4-*b*]pyridine-5-carbonitrile (9a).


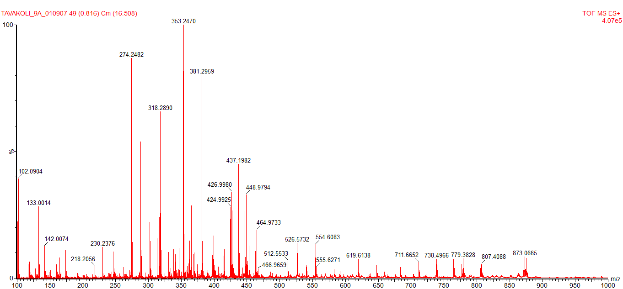


# Mass spectrum of 4-(4-bromophenyl)-3-(4-chlorophenyl)-6-oxo-6,7-dihydro-1*H*-pyrazolo[3,4-*b*]pyridine-5-carbonitrile (9a).


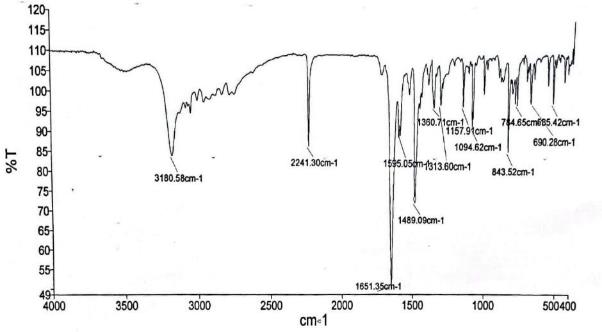


# FT-IR spectrum of 3,4-bis(4-chlorophenyl)-6-oxo-6,7-dihydro-1*H*-pyrazolo[3,4-*b*]pyridine-5-carbonitrile (10a).


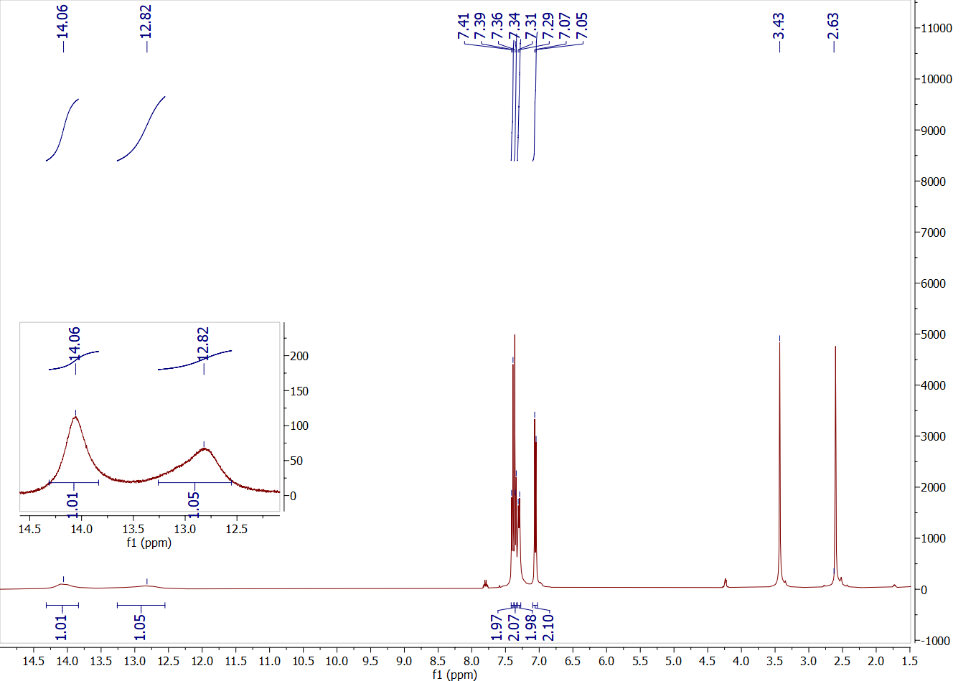


# ^1^H-NMR spectrum of 3,4-bis(4-chlorophenyl)-6-oxo-6,7-dihydro-1*H*-pyrazolo[3,4-*b*]pyridine-5-carbonitrile (10a).


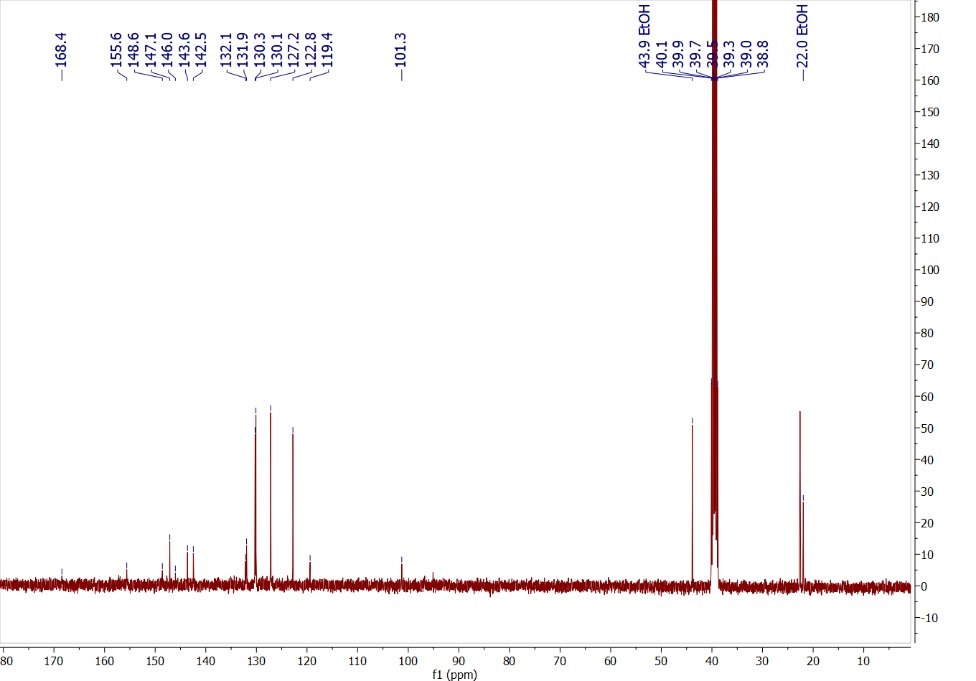


# ^13^C-NMR spectrum of 3,4-bis(4-chlorophenyl)-6-oxo-6,7-dihydro-1*H*-pyrazolo[3,4-*b*]pyridine-5-carbonitrile (10a).


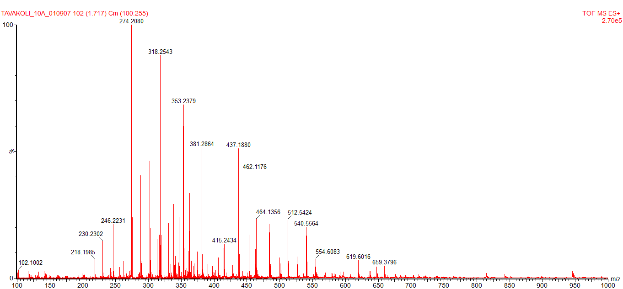


# Mass spectrum of 3,4-bis(4-chlorophenyl)-6-oxo-6,7-dihydro-1*H*-pyrazolo[3,4-*b*]pyridine-5-carbonitrile (10a).


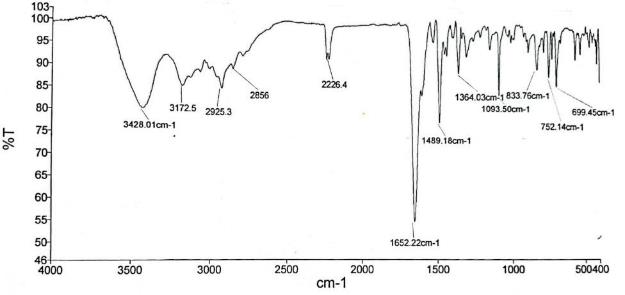


# FT-IR spectrum of 3-(4-chlorophenyl)-6-oxo-4-phenyl-6,7-dihydro-1*H*-pyrazolo[3,4-*b*]pyridine-5-carbonitrile (11a).


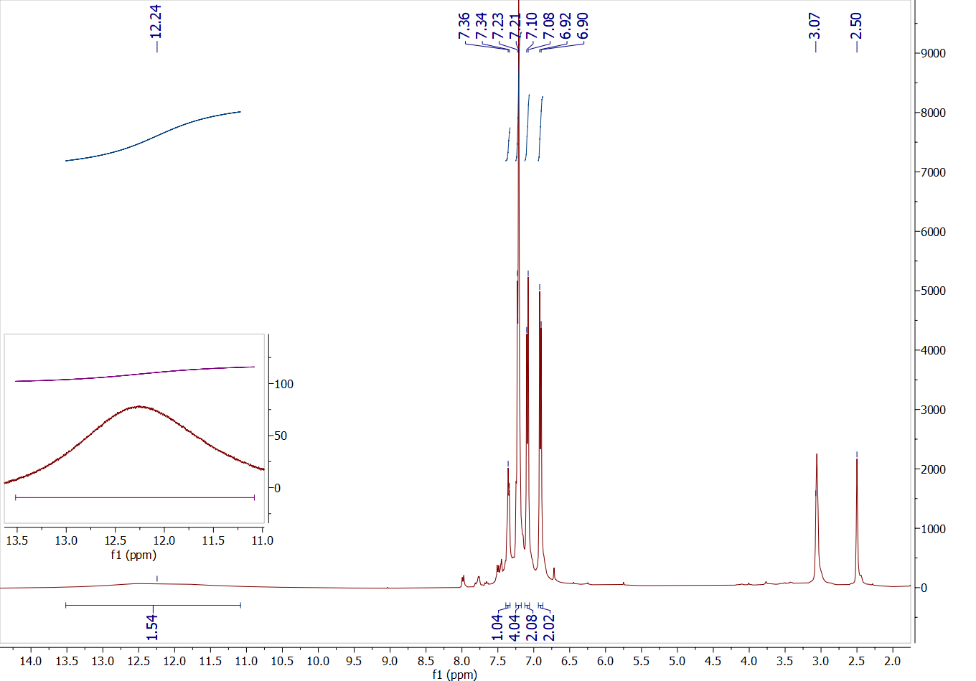


# ^1^H-NMR spectrum of 3-(4-chlorophenyl)-6-oxo-4-phenyl-6,7-dihydro-1*H*-pyrazolo[3,4-*b]*pyridine-5-carbonitrile (11a).


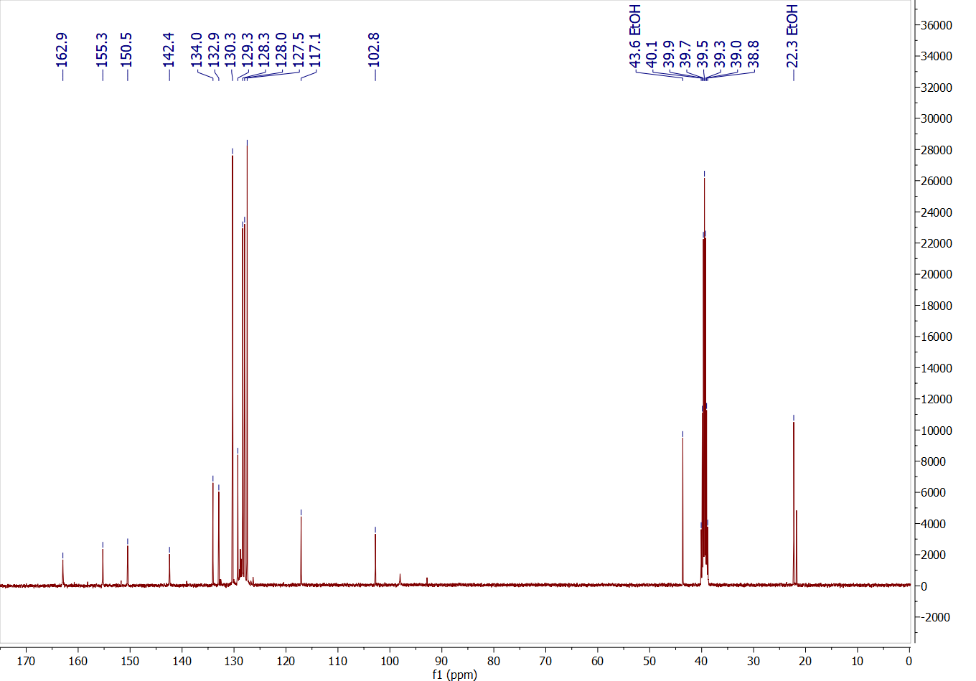


# ^13^C-NMR spectrum of 3-(4-chlorophenyl)-6-oxo-4-phenyl-6,7-dihydro-1*H*-pyrazolo[3,4-b]pyridine-5-carbonitrile (11a).


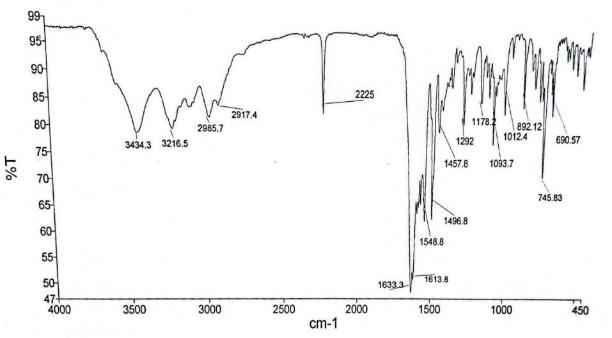


# FT-IR spectrum of 4-(4-chlorophenyl)-3-(1*H*-indol-3-yl)-6-oxo-1-phenyl-6,7-dihydro-1*H*-pyrazolo[3,4-*b*]pyridine-5-carbonitrile (1b).


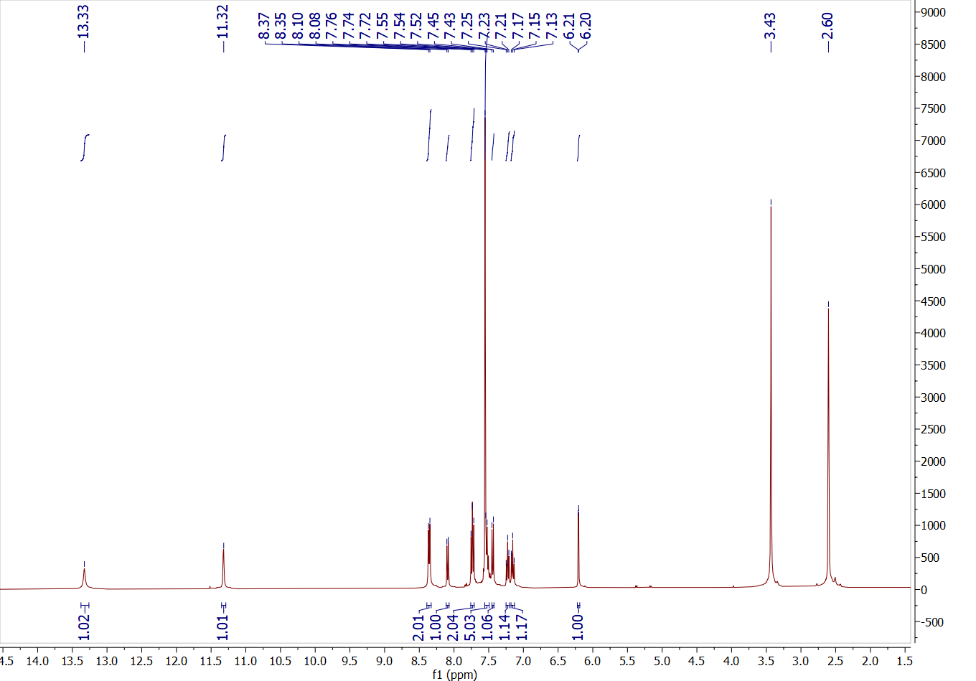


# ^1^H-NMR spectrum of 4-(4-chlorophenyl)-3-(1*H*-indol-3-yl)-6-oxo-1-phenyl-6,7-dihydro-1*H*-pyrazolo[3,4-*b*]pyridine-5-carbonitrile (1b).


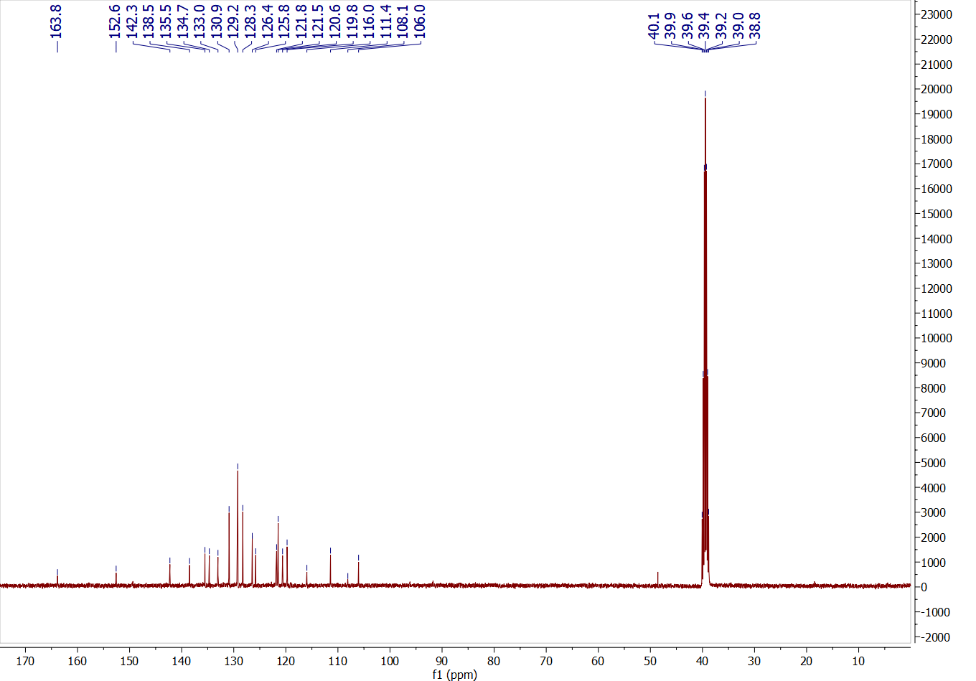


# ^13^C-NMR spectrum of 4-(4-chlorophenyl)-3-(1*H*-indol-3-yl)-6-oxo-1-phenyl-6,7-dihydro-1*H*-pyrazolo[3,4-b]pyridine-5-carbonitrile (1b).


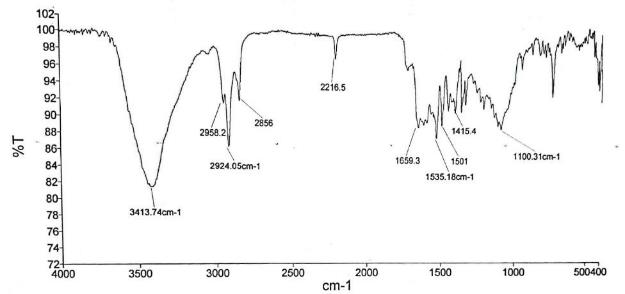


# FT-IR spectrum of 3-(1*H*-indol-3-yl)-4-(4-isopropylphenyl)-6-oxo-1-phenyl-6,7-dihydro-1*H*-pyrazolo[3,4-*b*]pyridine-5-carbonitrile (2b).


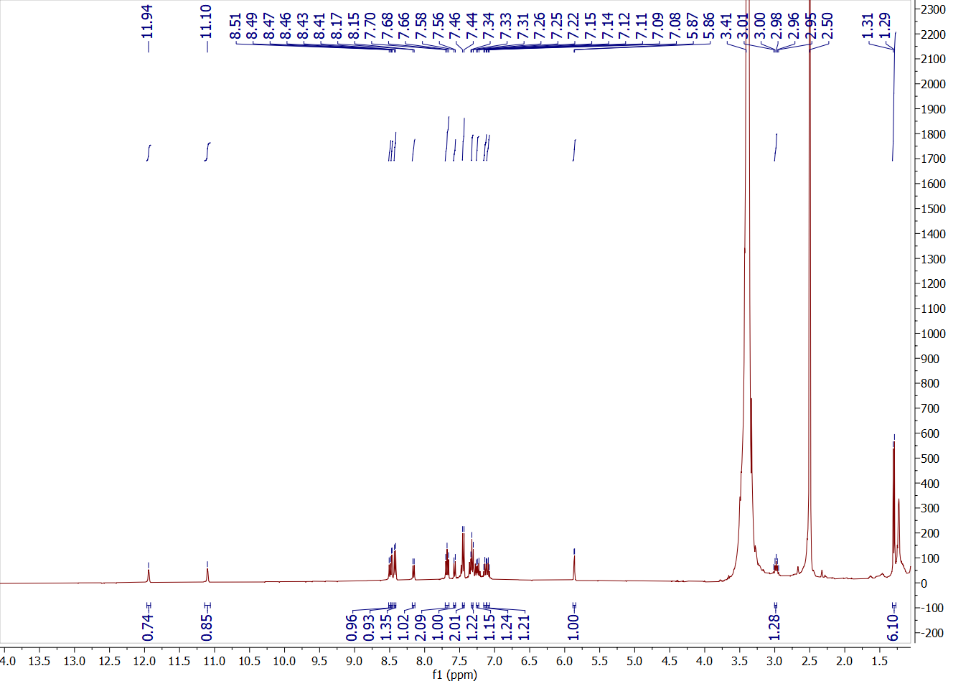


# ^1^H-NMR spectrum of 3-(1*H*-indol-3-yl)-4-(4-isopropylphenyl)-6-oxo-1-phenyl-6,7-dihydro-1*H*-pyrazolo[3,4-*b*]pyridine-5-carbonitrile (2b).


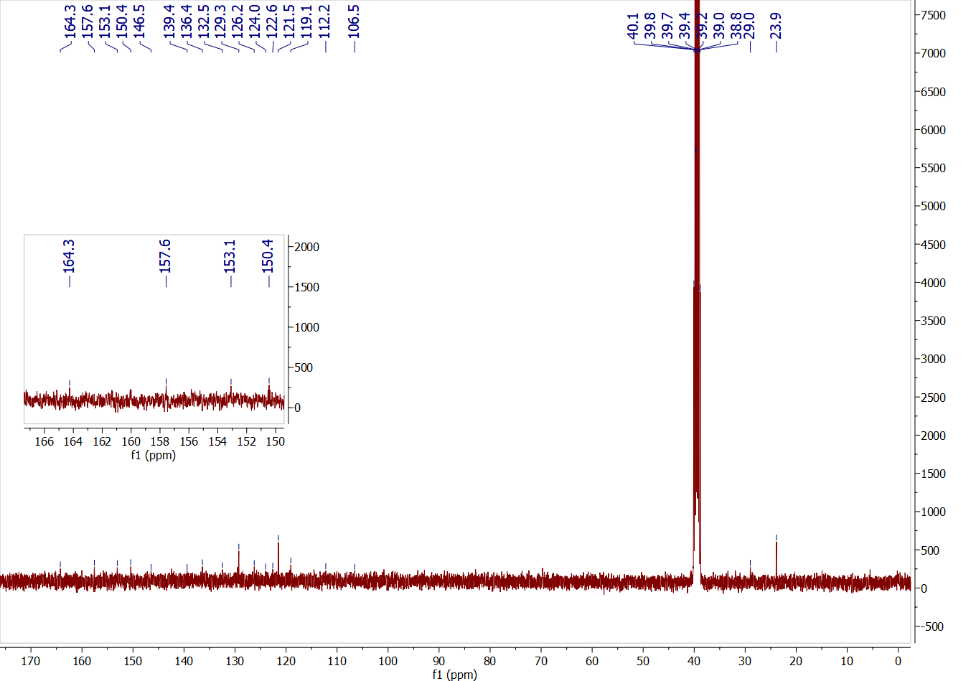


# ^13^C-NMR spectrum of 3-(1*H*-indol-3-yl)-4-(4-isopropylphenyl)-6-oxo-1-phenyl-6,7-dihydro-1*H*-pyrazolo[3,4-*b*]pyridine-5-carbonitrile (2b).


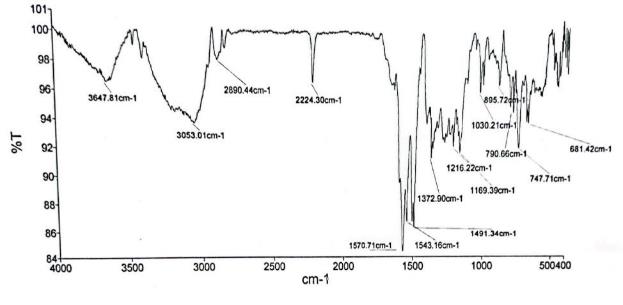


# FT-IR spectrum of 3-(1*H*-indol-3-yl)-6-oxo-1-phenyl-4-(pyridin-4-yl)-6,7-dihydro-1*H*-pyrazolo[3,4-*b*]pyridine-5-carbonitrile (3b).


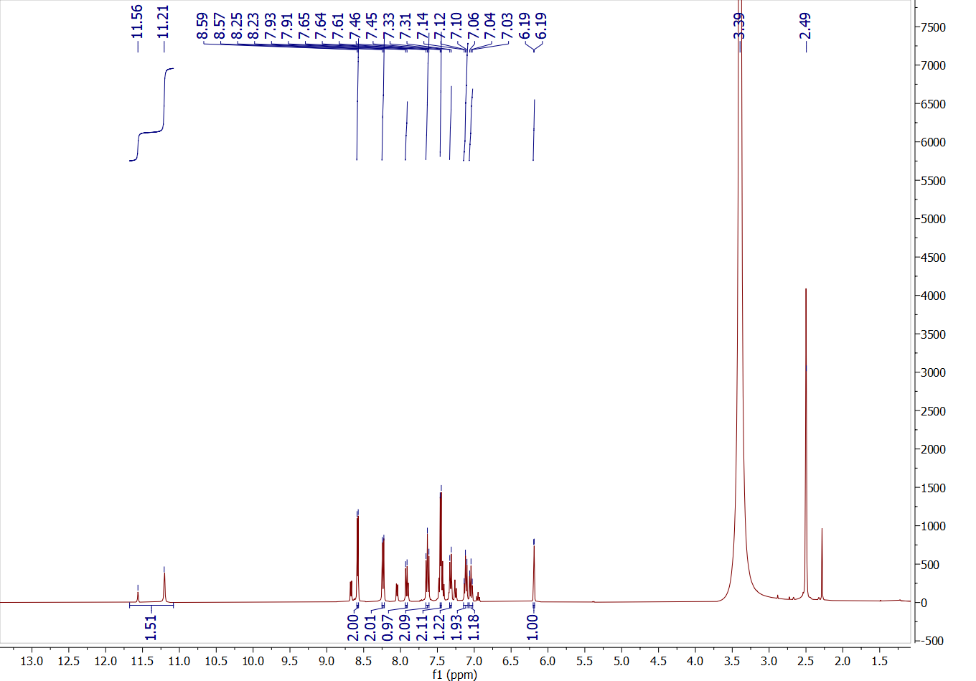


# ^1^H-NMR spectrum of 3-(1*H*-indol-3-yl)-6-oxo-1-phenyl-4-(pyridin-4-yl)-6,7-dihydro-1*H*-pyrazolo[3,4-*b*]pyridine-5-carbonitrile (3b).


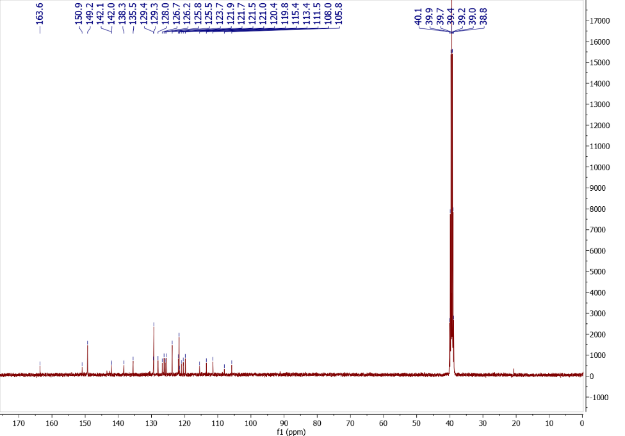


# ^13^C-NMR spectrum of 3-(1*H*-indol-3-yl)-6-oxo-1-phenyl-4-(pyridin-4-yl)-6,7-dihydro-1*H*-pyrazolo[3,4-*b*]pyridine-5-carbonitrile (3b).


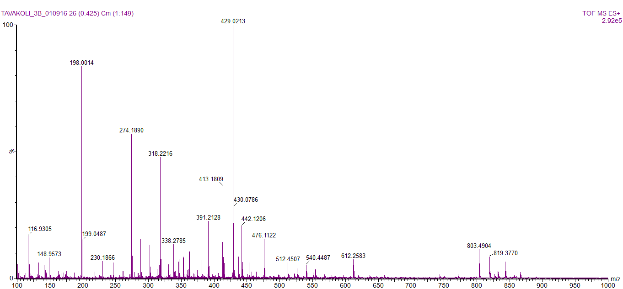


Mass spectrum of 3-(1*H*-indol-3-yl)-6-oxo-1-phenyl-4-(pyridin-4-yl)-6,7-dihydro-1*H*-pyrazolo[3,4-*b*]pyridine-5-carbonitrile (3b).


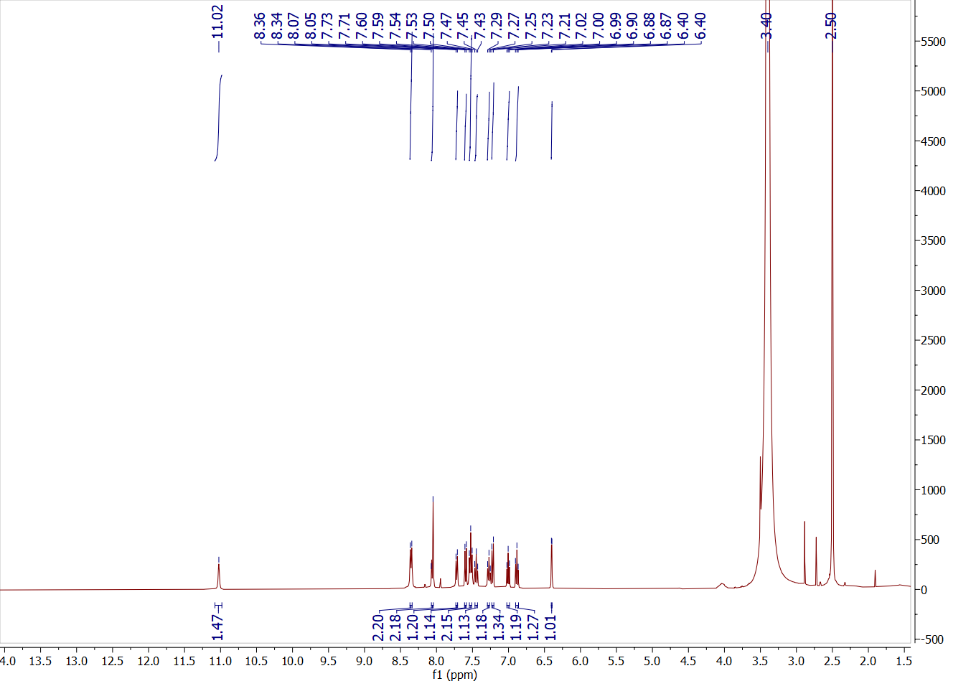


# ^1^H-NMR spectrum of 3-(1*H*-indol-3-yl)-4-(3-nitrophenyl)-6-oxo-1-phenyl-6,7-dihydro-1*H*-pyrazolo[3,4-*b*]pyridine-5-carbonitrile (4b).


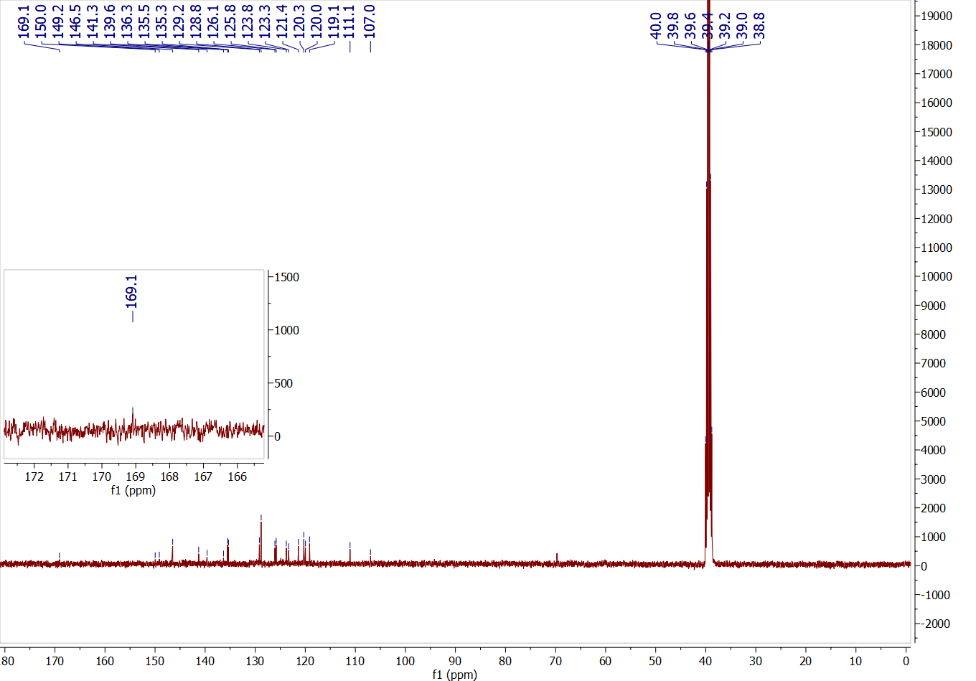


# ^13^C-NMR spectrum of 3-(1*H*-indol-3-yl)-4-(3-nitrophenyl)-6-oxo-1-phenyl-6,7-dihydro-1*H*-pyrazolo[3,4-*b*]pyridine-5-carbonitrile (4b).


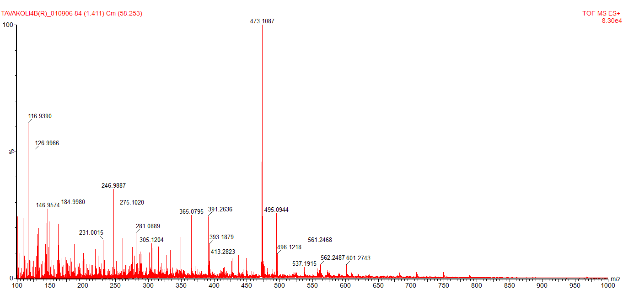


# Mass spectrum of 3-(1*H*-indol-3-yl)-4-(3-nitrophenyl)-6-oxo-1-phenyl-6,7-dihydro-1*H*-pyrazolo[3,4-*b*]pyridine-5-carbonitrile (4b).


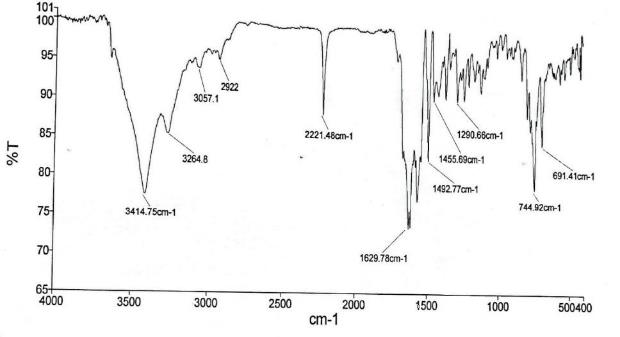


# FT-IR spectrum of 3-(1*H*-indol-3-yl)-6-oxo-1-phenyl-4-(*m*-tolyl)-6,7-dihydro-1*H*-pyrazolo[3,4-*b*]pyridine-5-carbonitrile (5b).


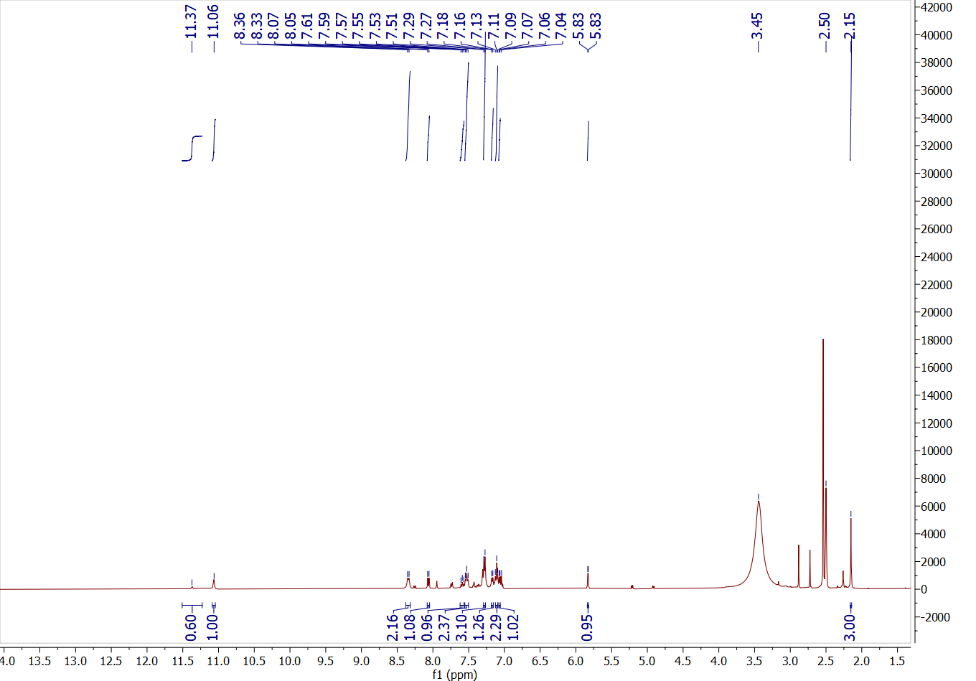


# ^1^H-NMR spectrum of 3-(1*H*-indol-3-yl)-6-oxo-1-phenyl-4-(*m*-tolyl)-6,7-dihydro-1*H*-pyrazolo[3,4-*b*]pyridine-5-carbonitrile (5b).


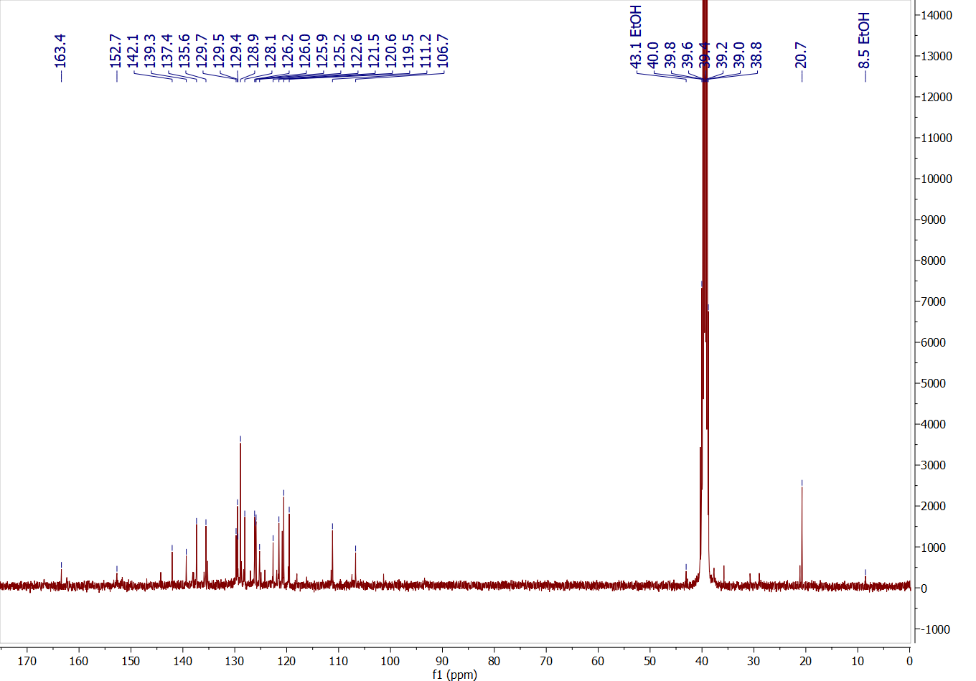


# ^13^C-NMR spectrum of 3-(1*H*-indol-3-yl)-6-oxo-1-phenyl-4-(*m*-tolyl)-6,7-dihydro-1*H*-pyrazolo[3,4-*b*]pyridine-5-carbonitrile (5b).


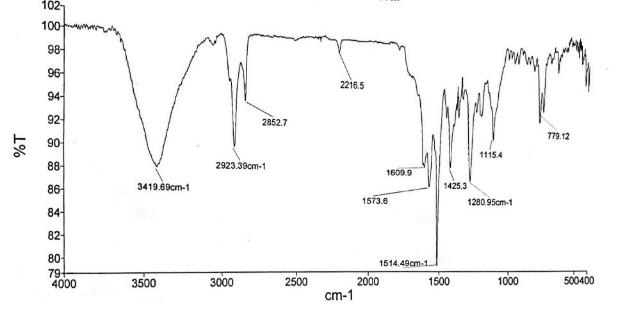


# FT-IR spectrum of 4-(2,4-difluorophenyl)-3-(1*H*-indol-3-yl)-6-oxo-1-phenyl-6,7-dihydro-1*H*-pyrazolo[3,4-*b*]pyridine-5-carbonitrile (6b).


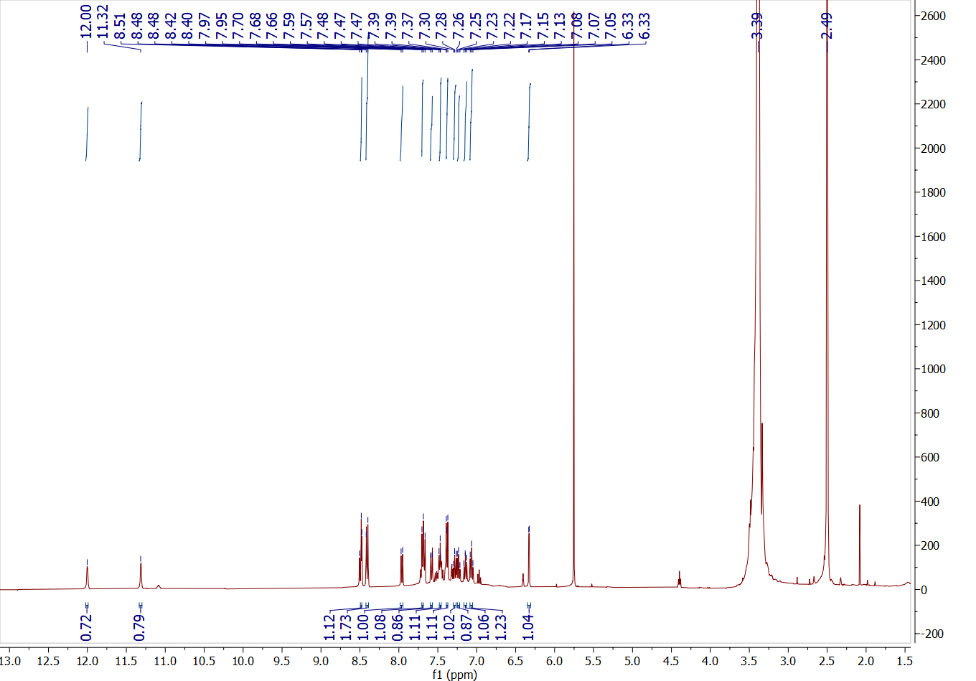


# ^1^H-NMR spectrum of 4-(2,4-difluorophenyl)-3-(1*H*-indol-3-yl)-6-oxo-1-phenyl-6,7-dihydro-1*H*-pyrazolo[3,4-*b*]pyridine-5-carbonitrile (6b).


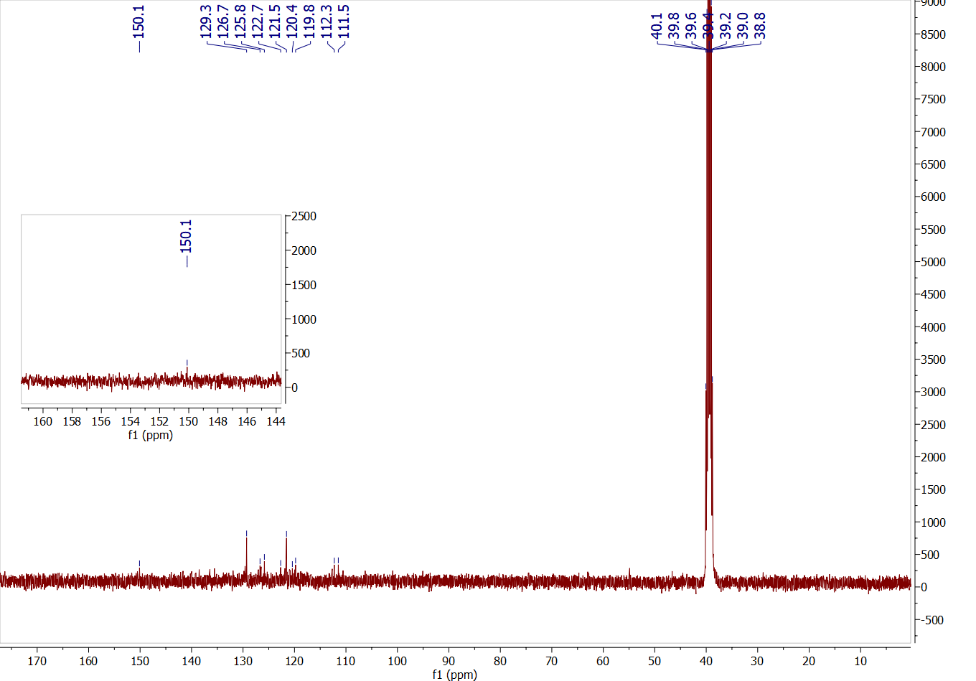


# ^13^C-NMR spectrum of 4-(2,4-difluorophenyl)-3-(1*H*-indol-3-yl)-6-oxo-1-phenyl-6,7-dihydro-1*H*-pyrazolo[3,4-*b*]pyridine-5-carbonitrile (6b).


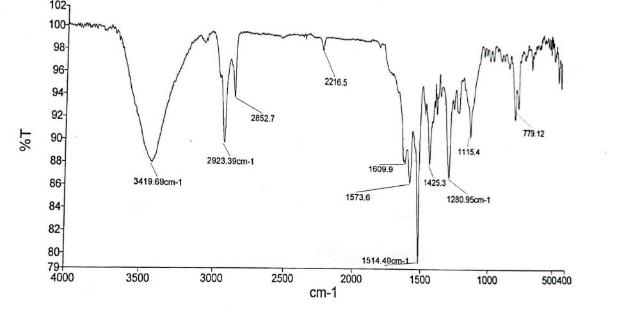


# FT-IR spectrum of 4-(4-bromophenyl)-3-(1*H*-indol-3-yl)-6-oxo-1-phenyl-6,7-dihydro-1*H*-pyrazolo[3,4-*b*]pyridine-5-carbonitrile (7b).


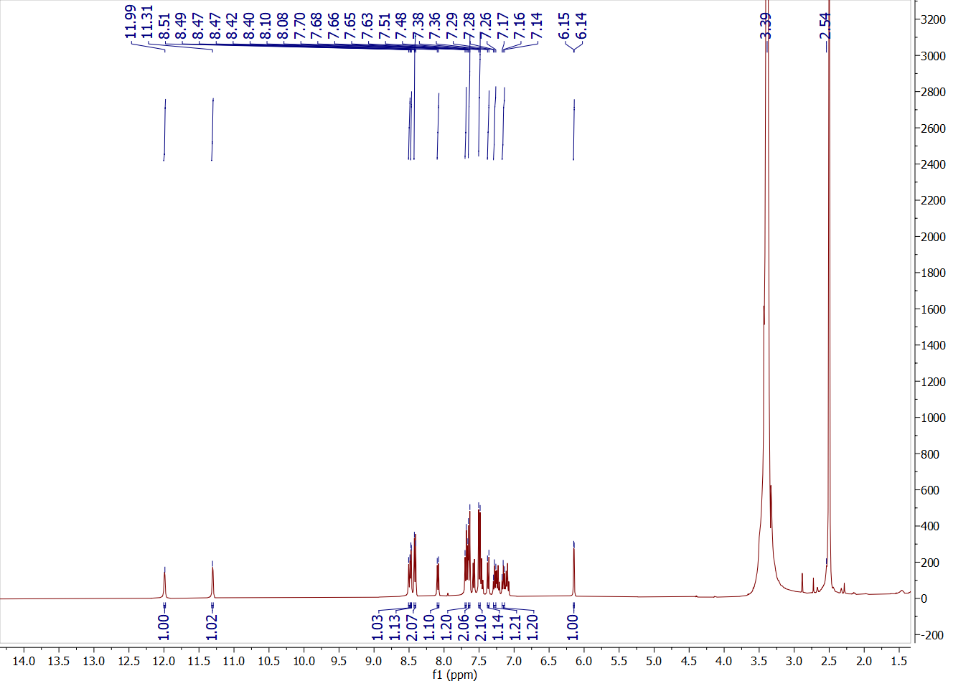


# ^1^H-NMR spectrum of 4-(4-bromophenyl)-3-(1*H*-indol-3-yl)-6-oxo-1-phenyl-6,7-dihydro-1*H*-pyrazolo[3,4-*b*]pyridine-5-carbonitrile (7b).


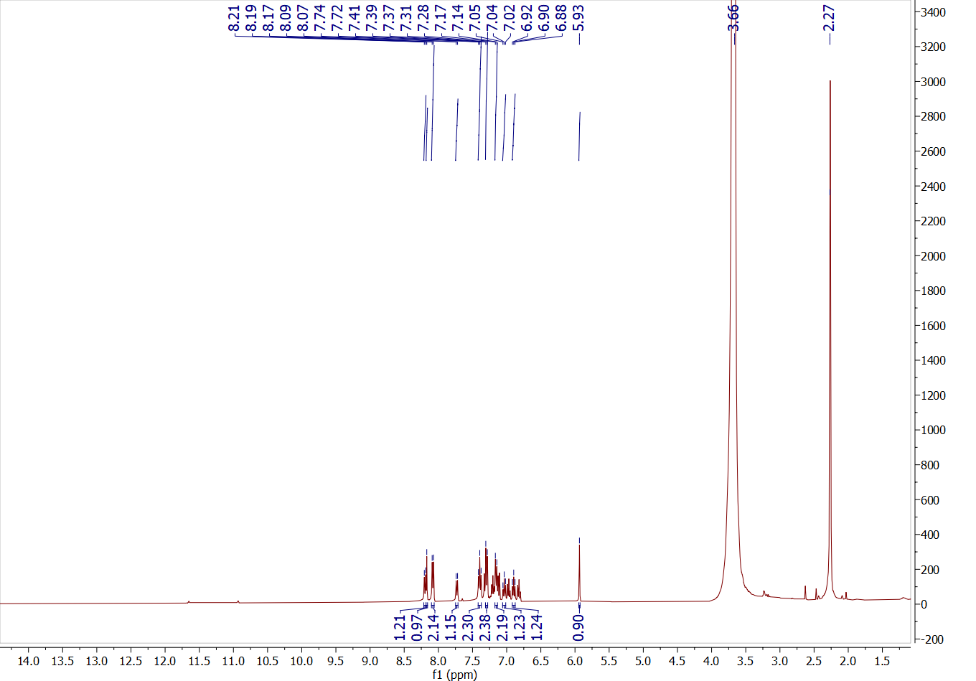


# D_2_O exchange spectrum of 4-(4-bromophenyl)-3-(1*H*-indol-3-yl)-6-oxo-1-phenyl-6,7-dihydro-1*H*-pyrazolo[3,4-*b*]pyridine-5-carbonitrile (7b).


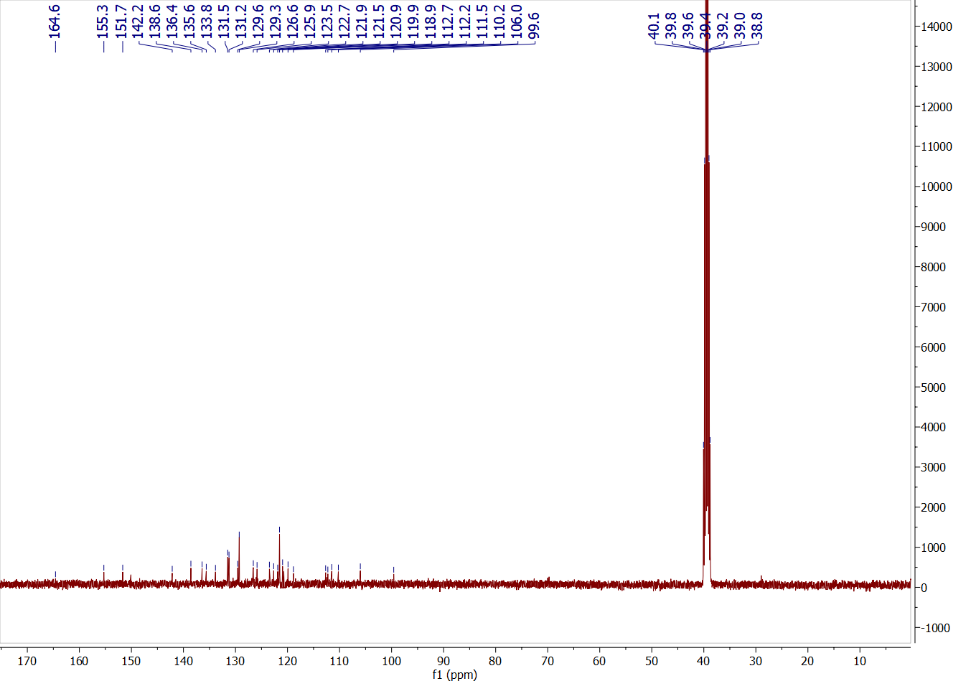


# ^13^C-NMR spectrum of 4-(4-bromophenyl)-3-(1*H*-indol-3-yl)-6-oxo-1-phenyl-6,7-dihydro-1*H*-pyrazolo[3,4-*b*]pyridine-5-carbonitrile (7b).


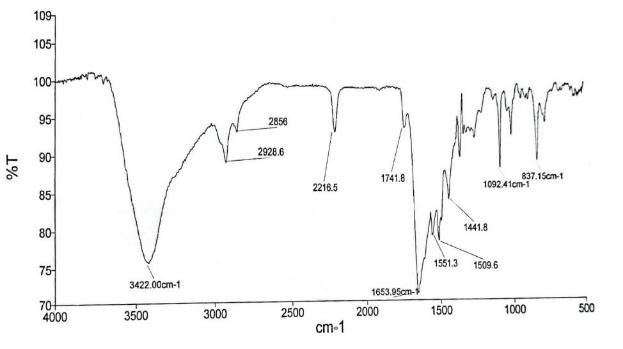


# FT-IR spectrum of 4,4'-(1,4-phenylene)bis(3-(1*H*-indol-3-yl)-6-oxo-1-phenyl-6,7-dihydro-1*H*-pyrazolo[3,4-*b*]pyridine-5-carbonitrile (8b).


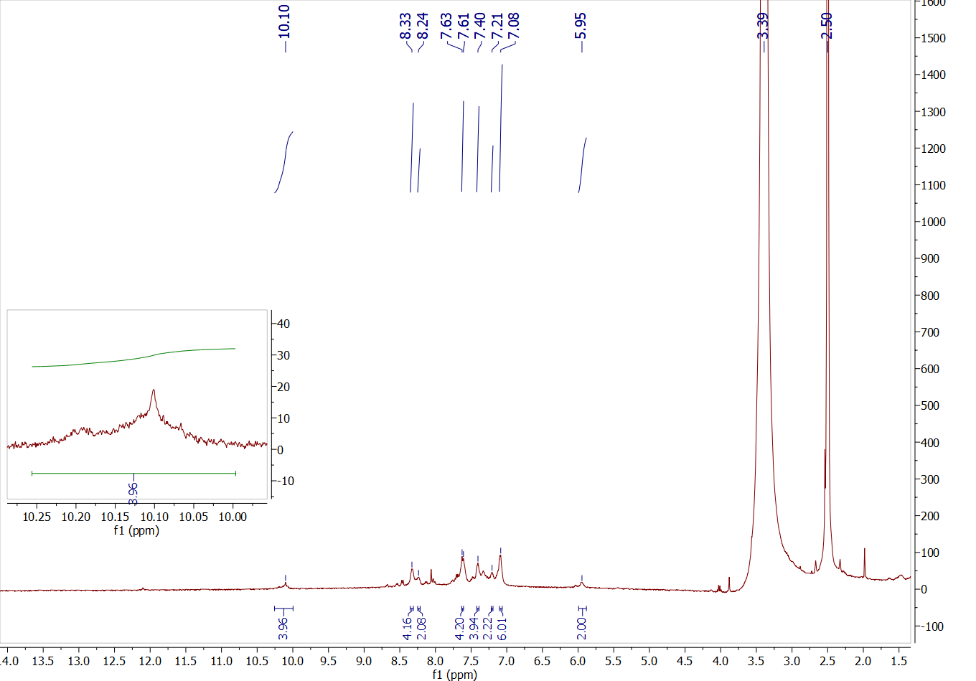


# ^1^H-NMR spectrum of 4,4'-(1,4-phenylene)bis(3-(1*H*-indol-3-yl)-6-oxo-1-phenyl-6,7-dihydro-1*H*-pyrazolo[3,4-*b*]pyridine-5-carbonitrile (8b).


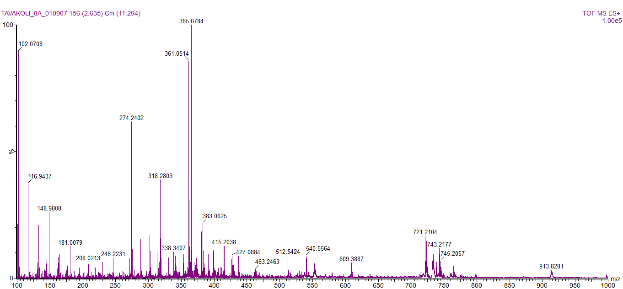


# Mass spectrum of 4,4'-(1,4-phenylene)bis(3-(1*H*-indol-3-yl)-6-oxo-1-phenyl-6,7-dihydro-1*H*-pyrazolo[3,4-*b*]pyridine-5-carbonitrile (8b).


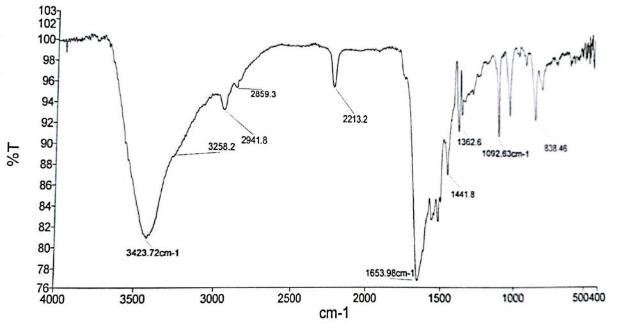


# FT-IR spectrum of 4,4'-(1,4-phenylene)bis(3-(4-chlorophenyl)-6-oxo-6,7-dihydro-1*H*-pyrazolo[3,4-b]pyridine-5-carbonitrile (12a).


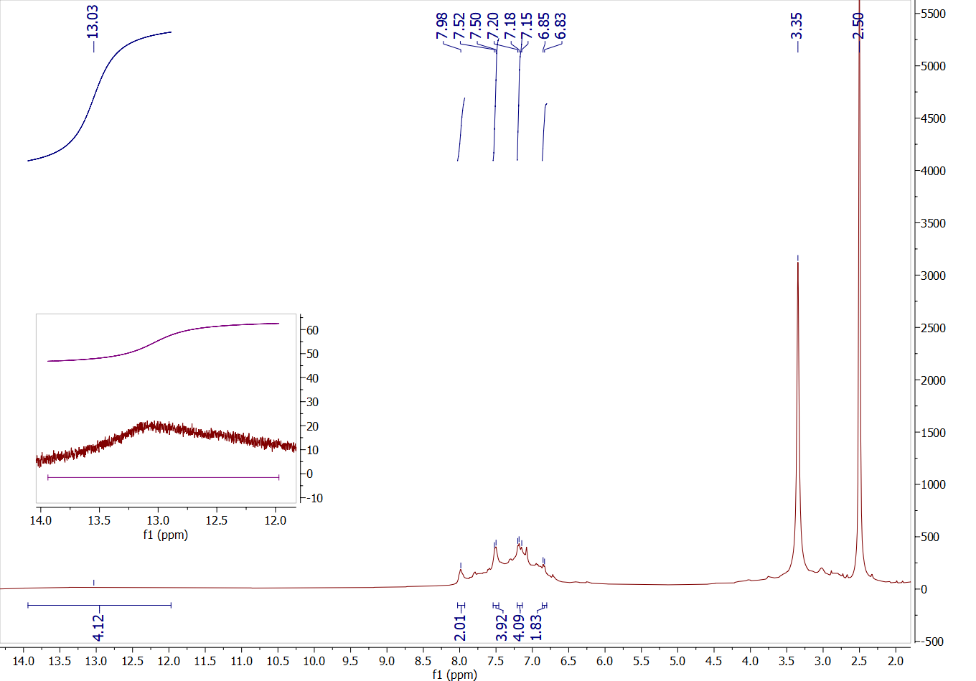


# ^1^H-NMR spectrum of 4,4'-(1,4-phenylene)bis(3-(4-chlorophenyl)-6-oxo-6,7-dihydro-1*H*-pyrazolo[3,4-*b*]pyridine-5-carbonitrile (12a).


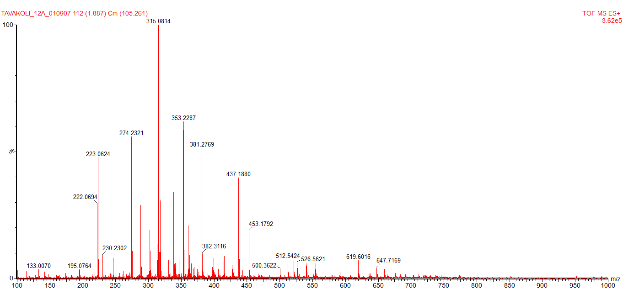


# Mass spectrum of 4,4'-(1,4-phenylene)bis(3-(4-chlorophenyl)-6-oxo-6,7-dihydro-1H-pyrazolo[3,4-b]pyridine-5-carbonitrile (12a).
